# A comprehensive narrative review on the significance of hyaluronic acid for dental implantology

**DOI:** 10.2340/biid.v13.45432

**Published:** 2026-02-27

**Authors:** Ahmed Yaseen Alqutaibi, Nazrah Maher, Anum Mahmood, Faiza Amin, Ghulam Irtiza Mustafa, Naresh Kumar, Muhammad Sohail Zafar

**Affiliations:** a Substitutive Dental Sciences Department (Prosthodontics), College of Dentistry, Taibah University, Madinah, Saudi Arabia; b Prosthodontics Department, College of Dentistry, Ibb University, Ibb, Yemen; c Department of Science of Dental Materials, Dow University of Health Sciences, Karachi, Pakistan; d Department of Clinical Sciences, College of Dentistry, Ajman University, Ajman, United Arab Emirates; e Centre of Medical and Bio-allied Health Sciences Research, Ajman University, Ajman, United Arab Emirates; f School of Dentistry, University of Jordan, Amman, Jordan

**Keywords:** Biomolecules, prosthodontics, biomaterials, coatings, scaffold

## Abstract

Dental implants have significantly advanced the scope of oral health care and practices, providing a stable and durable solution for replacing missing teeth. Essential maintenance practices, including regular oral hygiene and professional monitoring, are imperative to prevent complications such as peri-implant diseases, which can compromise implant integrity. Supplementary agents, including hyaluronic acid (HA), have been shown to enhance healing and integration. HA is recognised for its moisture-retaining properties, its promotion of wound healing, its reduction of inflammation, and its facilitation of tissue integration. The extensive therapeutic applications of HA in dental implant therapy are due to its biocompatibility and regulatory influences on cellular behaviour, which render HA a valuable adjunct to implant success, particularly concerning soft and hard tissue integration around the implants.

The present study aims to explore the potential applications of HA in dental implantology, including the modification of implant surfaces, the promotion of soft tissue healing around the implants, and the management of peri-implant diseases, such as mucositis and peri-implantitis. In addition, this study explores the role of HA in alveolar bone regeneration, particularly through alveolar ridge augmentation and preservation processes, as well as more advanced techniques such as tissue engineering, as the primary requirement for successful implant placement is sufficient bone width and depth. HA promotes osseointegration, increases osteogenesis, and helps treat peri-implant conditions such as mucositis and peri-implantitis. Implant biocompatibility, hydrophilicity, and bacterial adhesion resistance are all enhanced by HA coatings, which facilitate improved peri-implant bone and soft tissue healing. Stability and healing properties of HA can be increased by combining it with other biomaterials. Future studies should focus on enhancing HA’s mechanical properties, improving long-term bioactivity, and investigating synergistic biomaterial combinations; clinical trials are required to fully understand its potential in implant dentistry.

## Graphical abstract

**Figure:**
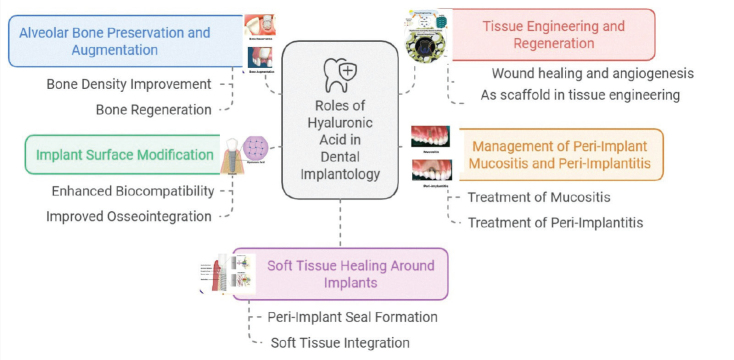
GRAPHICAL ABSTRACT

## Introduction

Dental implants have revolutionised modern dentistry by providing a predictable and durable solution for tooth loss that restores function, aesthetics, and quality of life [1, 2]. The procedure involves surgically placing the implant in the jawbone, and in straightforward cases, the surgery is relatively simple with a high success rate [3]. However, some cases require additional bone or soft tissue augmentation to ensure optimal conditions for implant integration and long-term stability [4]. Grafting is performed when there’s insufficient bone density or volume by using mineralised material, such as bone or hydroxyapatite, bioactive glasses, calcium carbonates, tricalcium phosphates, among others. At the same time, soft tissue augmentation improves the soft tissue at the implant site. These augmentations are essential for successful implant integration and aesthetic outcomes [5]. Supportive materials such as hyaluronic acid (HA), platelet-rich plasma, and bone morphogenetic proteins enhance healing and integration in bone grafting and implant procedures [6, 7]. PRF accelerates healing, promoting blood vessels and bone formation [7], while BMPs guide stem cells to form bone [8]. However, HA, a highly versatile macromolecule, offers distinct advantages over other biomolecules due to its unique and highly promising properties. Its high biocompatibility, biodegradability, and non-immunogenicity, coupled with the ability to retain moisture and mediate various cellular responses in the body, create a moisture-rich environment that supports both hard and soft tissue healing while minimising inflammation [9]. Furthermore, HA’s natural presence in the human body enhances its biocompatibility and reduces the risk of adverse reactions, making it a versatile and vital option for therapeutic applications [10, 11].

Owing to these properties, HA also performs a critical role in enhancing the outcomes of dental implant procedures [11]. In dental implantology, HA promotes wound healing [12], reduces inflammation [13], and supports both hard and soft tissue integration around the implant [14]. Its administration produces a moisture-rich environment that enhances soft tissue repair and healing, alleviates postoperative discomfort, and minimises the risk of complications such as infection [15]. Furthermore, HA’s role extends to improving the quality and volume of preimplant bone tissues, which is critical for achieving both functional and aesthetic outcomes in implant placement. By maintaining hydration and promoting cell migration, HA improves soft tissue integration, making it an essential adjunct in cases requiring soft tissue augmentation around implants. Therefore, HA is utilised at various stages of dental implant treatment due to its effects on both soft and hard tissue healing. To date, numerous clinical trials have also been conducted to explore the application of HA in dental implant treatment. Currently, no detailed narrative review has been published that examines all possible effects and applications of HA in dental implant therapy to improve clinical success and longevity.

Therefore, the purpose of this narrative review was to explore HA in the context of dental implant therapy, and the main objectives of our study are to:

1. Discuss all the potential applications of HA in dental implantology, including alveolar bone preservation, augmentation, management of peri-implant diseases, implant surface modification, soft tissue healing around implants, and tissue engineering for alveolar bone regeneration.
2. Discuss potential complications associated with HA applications alongside the proposed strategies for effectively managing these issues.

For this narrative review, three electronic databases – Google Scholar, Science Direct, and PubMed – were used to search the articles by using the following keywords: ‘hyaluronic acid’, ‘dental implant therapy’. The search was limited to studies in English. Those articles that investigated the potential application of HA in wound repair, tissue engineering, alveolar bone preservation (ARP) and augmentation, and implant procedures were selected for this study. Thus, the data for this review article have been retrieved from original research studies, systematic analysis, and review papers.

## Discussion

### History of hyaluronic acid

**Figure 1:**
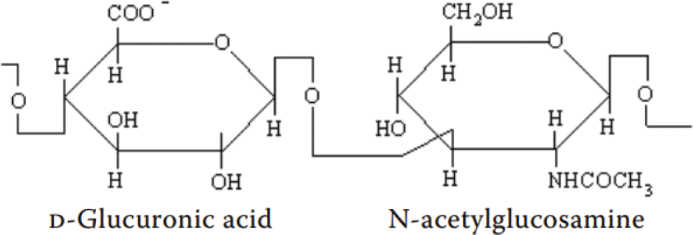
Chemical structure of hyaluronic acid [22].

**Figure 2:**
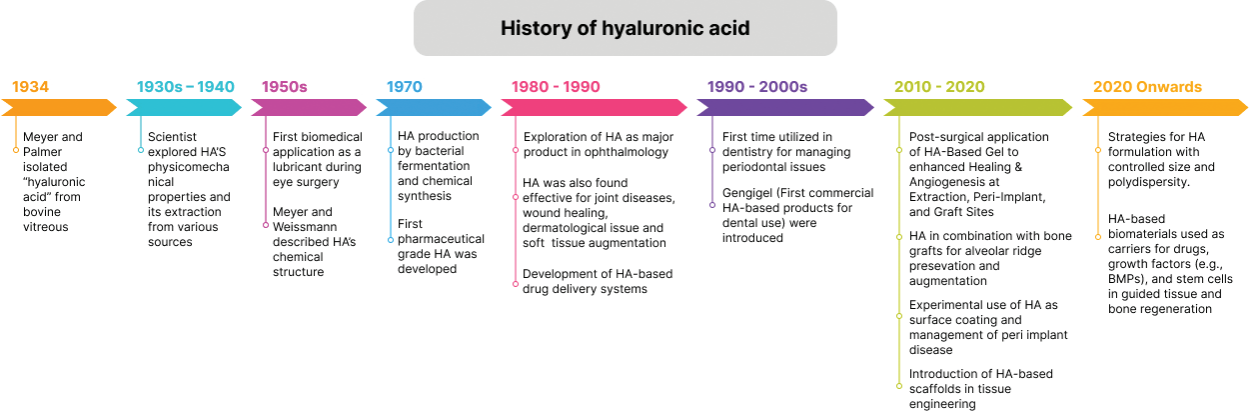
The progressive development of hyaluronic acid (HA) over the years.

The first study documenting HA dates back to 1880, when Portes, a French scientist, observed that the vitreous body mucin differed from the other mucoids present in the cornea and cartilage and named it ‘hyalomucine’ [16]. However, it was in 1934, two German biochemists, Karl Meyer and John Palmer, extracted a new polysaccharide from the vitreous humor of the cow and called it ‘hyaluronic acid’, derived from the words ‘hyaloid’ (clear and glass-like appearance) and uronic acid [17]. During the 1930s – 1950s, researchers explored HA’s physicomechanical properties [18, 19] as well as its isolation from various sources, and the primary sources of extraction were rooster comb, streptococci, and human umbilical cord [20]. In 1954, Meyer and Weissmann solved and described the chemical structure of HA for the first time: it is a straight-chain polymer comprising two alternating sugars D-glucuronic acid and N-acetyl-D-glucosamine (Figure 1) [21]. The initial studies on HA synthesis through bacterial fermentation and chemical production were also conducted during this period [16]. The first biomedical application of HA occurred in the late 1950s when it was utilised as a lubricant during eye procedures [22]. However, Balazs pioneered the effective extraction and purification of pharmaceutical-grade HA from rooster combs and human umbilical cords in 1979, laying the foundation for industrial production [23]. From the early 1980s, HA was explored and utilised as a significant product in ophthalmology, including vitreous replacement during eye surgery, corneal hydration, dry eye treatment, among others [24, 25]. It was also found to be especially useful in cataract surgery, where it helped maintain the eye’s viscosity and moisture stability during the procedure [24]. Furthermore, HA was then also discovered to be effective for the treatment of joint diseases [26] and dermatological issues [27], wound repair [28], and soft tissue augmentation [29]. During the late 1980s and 1990s, HA was employed to develop drug delivery systems, and efforts continue to produce HA-based carriers to improve therapeutic efficacy [30, 31]. HA was also first introduced in dentistry for treating periodontal diseases, as a clinical trial conducted in 1977 demonstrated that HA exhibited anti-inflammatory, anti-oedematous, and anti-bacterial effects for managing periodontal conditions [32]. Since then, from 2010 to 2020, HA has been utilised for various dental applications, including HA-based gel to improve healing outcomes after dental procedures, HA in combination with bone grafts for bone regeneration in ARP and augmentation procedures, and implant surface modification for improved osseointegration [33–35]. In the 2000s, special focus was also given to identifying and characterising the enzymes involved in HA metabolism, as well as developing bacterial fermentation strategies to manufacture HA with controlled size and polydispersity [16]. Also, the HA-based scaffolds were introduced in tissue engineering which offer a bioactive scaffold, effective in both its full and degraded length forms, and the cell receptor interactions with the scaffold can also be tailored to promote tissue growth and repair [36]. The work on HA in tissue engineering is ongoing, with a special focus on using 3D bio-printing for scaffold fabrication [37]. HA is currently a critical chemical for biomedical applications, therefore research continues to focus on better understanding its biosynthesis and molecular biology, improving biotechnological production, developing derivatives with superior characteristics, and refining its medicinal applications. Figure 2 shows the progressive development of HA over the years.

Various HA formulations, as shown in Table 1, are utilised in dentistry, including powder gel, spray, gel, and mouthwash, which enhance healing by promoting bone growth, reducing inflammation, managing swelling, and improving postoperative comfort and wound healing.

**Table 1:** Available HA formulations for dental applications.

Hyaluronic acid formulations	Description	References
HA-based Powder Gel	A formulation (200 micrograms) combining porous spherical particles of recombinant human bone morphogenetic protein-2 (rhBMP-2) with β-tricalcium phosphate microspheres. Facilitates the delivery of rhBMP-2 at bone defect sites, promotes new bone growth, and enhances osseointegration.	[38]
Spray	Effective for immediate postoperative use after dental extractions. Helps manage swelling and trismus with higher patient satisfaction compared to gel (0.2%) due to ease of application. Improves healing and postoperative comfort following impacted third molar surgery.	[39–41]
Gel	Facilitates tissue repair and healing. Reduces inflammation and IL-1 β levels in peri-implant pockets, and promotes faster healing after laser surgery.	[41]
Mouthwash	Convenient for post-surgical healing when applying topical treatments is painful. Reduces post-surgical edema, plaque, and bleeding. Promotes wound healing in surgical sites when combined with chlorhexidine (CHX).	[42]

### Potential applications of HA in dental implantology

#### HA in alveolar bone preservation and augmentation

Implant insertion in the alveolar bone requires sufficient bone depth and width to accommodate the supportive implant crown, which replaces the missing tooth and provides anchorage and optimal functionality while minimising movement and the potential for failure [43]. ARP and augmentation are critical dental procedures aimed at maintaining and rebuilding the jawbone. The primary objective of the ARP procedure is to reduce bone loss after extraction and to preserve the original bone volume and contour for future implant placement. This procedure employs a variety of materials, including bone grafts, bone substitutes, membranes, and haemostatic agents (such as gelatine sponge, collagen membranes, and platelet-rich plasma), to fill the extraction sockets immediately after the tooth extraction [44]. In contrast, alveolar bone augmentation is a surgical procedure designed to increase bone volume in the jaw prior to implant placement [45]. This procedure is typically performed when the existing bone is insufficient to support dental implants due to significant ridge resorption. Ridge augmentation can be achieved through guided bone regeneration, sinus lift procedures, bone grafting, and ridge-splitting techniques [46, 47]. While the primary goal of both procedures is to maintain bone mass, preservation is considered a preventive approach that halts bone loss. In contrast, augmentation is a curative approach that seeks to replace missing bone.

Due to their osteoconductive and osteoinductive properties, autogenous grafts are considered the gold standard for managing ARP and augmentation [2]. Currently, the use of grafts with bioactive components that can modulate intra- and extracellular responses to accelerate osteogenesis is receiving tremendous attention [48]. Among these, HA, as a bioactive molecule, has also been utilised to promote bone regeneration. *In vitro* experiments have demonstrated that HA enhances bone formation and remineralisation by stimulating mesenchymal cell differentiation, proliferation, migration into the osteoblastic lineage, angiogenesis, and the release of growth factors. In addition, data indicate that HA-based microparticles can covalently bond to the metal surfaces of implants and release biologically active constituents, thereby improving osteogenic processes and bone-to-implant integration [49]. However, the structure of HA disintegrates rapidly in the biological environment through hydrolysis. Consequently, composites of HA with other bone-grafting biomaterials (e.g. bovine bone, sticky bone) are typically used to extend the presence of HA over a longer period to enhance bone healing. Therefore, HA-based composites have also demonstrated successful results in bone remineralisation and osteogenesis [50].

**Figure 3:**
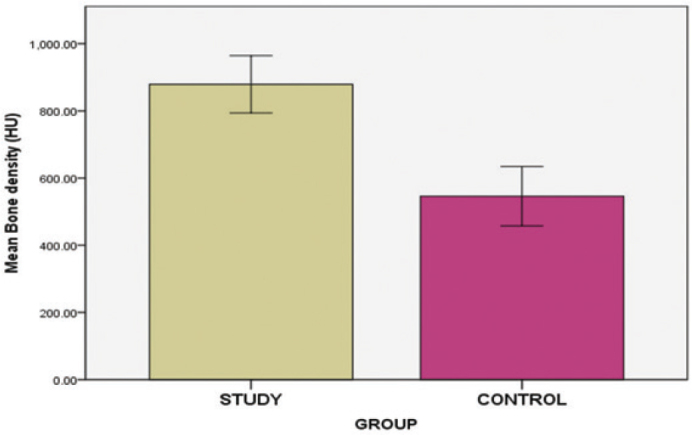
Bone density found for the study group (HA incorporated graft) and the control group (without HA incorporated graft) [52]

**Figure 4:**
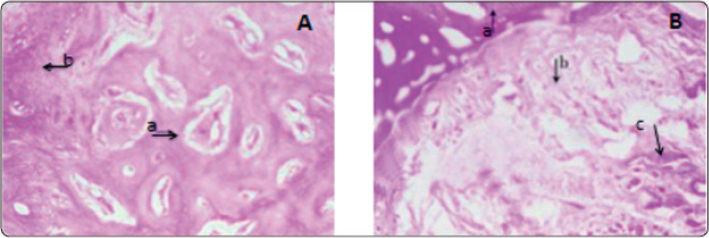
(A) Histological presentation of alveolar bone socket after placement of Osteon II collagen (30% beta tricalcium phosphate and 70% hydroxyapatite) mixed with HA at 6-months, showing (a) well-developed trabecular bone and (b) a small segment of fibrous tissues. (B) Histological presentation of the alveolar bone socket after placement of sticky bone graft at 6 months showing (a) newly formed bone, (b) thick and compact fibrous tissues, and arrow (c) residual grafting material. This indicates that the alveolar bone socket preserved with a combination of Osteon and hyaluronic acid showed a higher amount of mature bone (A), while the alveolar bone socket preserved with only sticky bone graft showed the highest amount of fibrous tissue and lower mature bone. (100 X magnification was utilised) [53].

A randomised clinical trial employed HA-incorporated demineralised bovine bone and evaluated both linear and volumetric bone resorption using cone beam computed tomography (CBCT) 4 months post-operatively, before implant placement. The findings showed that the HA reinforced group significantly (*P* = 0.018) limited post-extraction resorption to a greater extent than the control group [51]. Another study assessed bone density after using a combination of HA and bone grafts, yielding similar results using cone beam computed tomography (CBCT) (Figure 3) [52]. One clinical trial compared the effects of osteon II collagen (alloplastic material composed of 30% betatricalcium phosphate and 70% hydroxyapatite) mixed with HA against a sticky bone graft for the preservation of the alveolar socket through histological evaluation. Microscopic histological examination revealed that the HA group exhibited the highest amount of mature compact and spongy bone, with a well-developed Haversian system. In contrast, the other group displayed delayed ossification and a greater amount of fibrous tissue (Figure 4) [53].

A systematic review conducted by Ronsivalle in 2025 [54] investigates the regenerative abilities of HA in alveolar ridge preservation, analysing its role in reducing bone resorption and supporting bone regeneration when used in combination with xenografts following tooth extraction. From 2012 to 2024, studies were searched and analysed, resulting in the inclusion of four studies in this review. All studies indicate that HA when combined with xenografts, has the potential to reduce bone resorption, increase bone volume and density (promoting greater bone formation), and improve graft stability compared to controls. Only one study shows no difference in wound repair and patient-reported outcomes between the control and HA-modified groups [54]. In 2025, Helal et al. [55] conducted a split-mouth randomised clinical trial in which 10 patients with bilateral posterior atrophic mandible (20 sides) were randomised to one of two groups using a 1:1 allocation ratio. Group I used a prefabricated computer-aided design (CAD)-computer-aided manufacturing (CAM) allogeneic bone block that had been hydrated with HA to increase the ridge, while Group II used saline. Group I had the highest bone gain and growth, according to CBCT analysis, and histological data likewise demonstrated better osseointegration, bone regeneration, and elevated expression of Cox-2, Osteopontin (OPN), and Vascular Endothelial Growth Factor (VEGF) [55].

Table 2 provides a detailed description of various studies and clinical trials that have employed HA either alone or in conjunction with bone grafts for preservation and augmentation of the alveolar ridge.

**Table 2:** Studies which have utilised HA for alveolar bone preservation and augmentation.

Study design	Objective of the studies	Material used	Results	References
Randomised controlled clinical trial	Evaluation of the effectiveness of HA-reinforced osteon II collagen and sticky bone for alveolar bone preservation.	HA-reinforced osteon II collagen and sticky bone.	Osteon II with the HA group showed higher bone density on CBCT and greater mature bone formation on histomorphometric analysis.	[53]
Retrospective study	Evaluation of the effectiveness of granular allografts for alveolar ridge preservation with and without the addition of HA.	Allogeneic bone grafts with HA.	HA-added allograft showed less vertical bone loss, graft shrinkage, and greater bone density.	[11]
Pilot study (Double blinded randomised clinical trial)	Evaluation of the efficacy of cross linked HA in DBBM (demineralised bovine bone material) for alveolar ridge preservation.	Cross-linked HA and DBBM.	Radiological finding: HA-reinforced DBBM presented less linear and volumetric bone resorption compared to control sites. Histological finding: HA-reinforced group resulted in greater osteogenic activity(new bone formation) and higher incorporation of the graft material in the newly formed bone.	[51]
Randomised controlled clinical trial	Comparison of the efficacy of HA against I-PRF (injectable platelet-rich fibrin) in combination with xenografts for alveolar ridge preservation.	HA and I-PRF in combination with xenografts.	Radiological finding: CBCT showed that the HA group presented the highest bone gain and lowest crestal bone loss. Histological finding: The HA group showed greater bone formation and less residual graft material on histomorphometric analysis.	[56]
Randomised controlled clinical trial	Evaluating the efficiency of a combination of HA and xenografts for post-extraction alveolar socket bone preservation.	HA with xenografts.	Radiological finding: HA group showed higher bone density and less bone resorption.	[52]
*In vivo* experimental study	Assessment of HA-dipped collagen-containing deproteinised bovine bone DBBM-C for preserving alveolar bone in compromised sockets.	HA with DBBM-C.	Radiological finding: Micro CT analysis revealed higher bone volume density in DBBM-C + HA group. Histological finding: HA-reinforced group showed higher osteoid formation and trabecular pattern. Clinical findings: No signs of complication were noted in HA reinforced groups.	[57]
Experimental study (*in vitro* and *in vivo*)	Development of novel HA/gelatine hydrogel-based scaffold/plug to provide haemostasis and bone regeneration in one-step alveolar socket preservation.	HG/TCP/BCP scaffold (HA-gelatine hydrogel (HG), β-tricalcium phosphate and biphasic calcium phosphate).	*In vitro* experiments findings: The HA-based plug was found to be biocompatible and could increase the expression of genes that promote osteogenesis (RUNX2, COL1, ALP, and OPN). *In vivo* experiments findings: The HA-based plug encouraged haemostasis and osteogenesis at the site of a bone defect in a rabbit model.	[58]
Clinical case report	A novel hyaluronate incorporated xenegenic bone graft (cerabone) coupled with maxgraft (allogeneic bone particle), for treating an alveolar ridge deficiency.	HA with allogeneic bone granules.	Radiological finding: The radiograph demonstrated complete osseointegration of implants 3 years postoperatively. Clinical finding: The patient was followed up for 3 years, and excellent soft and hard tissue healing was observed without complications.	[59]
Randomised controlled clinical trial	Assessment of the combined effects of melatonin and hydroxyapatite (HA) for bone regeneration in maxillary sinus augmentation.	HA and melatonin.	An increase in bone density and vertical bone height was observed in the HA-melatonin group.	[60]
*In vivo* experimental study	Evaluation of the effectiveness of DBBM coupled with high-molecular-weight HA for bone regeneration.	HA combined with DBBM.	Radiological finding: Micro CT analysis showed higher trabecular bone formation in HA reinforced group. Histological finding: The HA group presented with angiogenesis and higher mature bone formation.	[61]

HA: hyaluronic acid; CBCT: .

### HA in the management of peri-implant diseases

#### HA in the management of peri-implant mucositis

Localised plaque accumulation around dental implants is recognised as the primary predisposing risk factor for implant-site infection, peri-implant mucositis (PiM), and peri-implantitis [62]. PiM is characterised by peri-implant soft tissue inflammation with no accompanying loss of bony tissues, resulting from the gradual and progressive build-up of plaque in the peri-implant sulcus [63, 64]. According to the Consensus Report from the workshop on the Classification of Periodontal and Peri-implant Diseases in 2017, PiM is diagnosed and characterised clinically by symptoms including redness, oedema, presence of exudate, and the BOP (bleeding on probing), without an increase in probing depth (PD) [65]. Scientific evidence indicates that PiM should be managed in its initial stages, as untreated mucositis can progress to peri-implantitis, leading to the progressive deterioration and loss of alveolar bone around the implant [66]. The European Federation of Periodontology [67] has established guidelines for reducing the risk of peri-implant-related infection, which recommend that the health status of the patients’ soft tissues around the implants be evaluated at each visit by measuring PD and BOP [68]. Intraoral radiography should be performed only when increased bleeding and PD are observed alongside suppuration [69]. Upon diagnosing PiM, appropriate treatment is necessary to prevent its progression to implantitis. Common treatment strategies for mucositis include plaque removal through scaling and root planing, followed by measures to control plaque [69, 70]. Latest research experiments have explored the application of topical and nebulised HA for managing PiM, owing to its antibacterial effects on periodontal bacterial pathogens [71], its ability to mediate the body’s innate response to microbes, and its significant role in wound healing by enhancing neo-angiogenesis and the attachment differentiation, and growth of cells involved in tissue repair [72, 73]. Moreover, HA has already been utilised in various clinical studies to treat or manage periodontal bony pockets, furcation defects, and gingival recession [74, 75]. These attributes support its application for the therapeutic management of PiM.

A recently published double-blind, randomised clinical trial conducted by Siciliano et al. [74] in 2024 assessed the clinical outcomes of patients with PiM following treatment with a topical HA gel 3 months post-operatively. Both the test and control groups received non-surgical debridement; however, only the test groups received a local topical gel consisting of sodium hyaluronate, spermidine, and sodium alginate. Bleeding during probing was evaluated as the main output of the study, alongside the other clinical parameters, including PD, full mouth plaque and bleeding score (FMPS and FMBS). The findings indicated significant improvements across all parameters after 3 months, with 85% of test implants demonstrating disease resolution compared to 70% in the control group [76].

Another clinical pilot study by Lopez et al. [75] evaluated the possible efficacy of nebulised HA in the management of mucositis after 15 days of application. The findings revealed that the difference in pocket depth between 0 and 15 days was not statistically significant. However, probing showed a noticeable decrease in bleeding at both the test and control sites, with greater improvement observed at the HA-treated sites [75].

These investigations suggest that topical HA application has potential for the non-surgical management of mucositis, particularly for limiting and addressing the condition in its early stages.

### HA in treating peri-implantitis

Dental implants are considered the primary choice for replacing missing teeth. However, 5–11% of cases may experience implant loss due to secondary failure [61]. Peri-implantitis is one of the leading causes of implant failure, with an estimated incidence rate of approximately 22%, which is estimated to increase in the coming years [77]. It is a deteriorative inflammatory pathology affecting both the soft and hard tissues encircling an already integrated implant. The condition is characterised by the advancing inflammation of the soft connective tissues and progressive loss of supporting bone [78]. Clinical indicators of peri-implantitis include radiographic bone loss, increased PD, and visible signs of inflammation, all of which can be observed at affected sites [79].

The treatment and management of peri-implantitis continue to be a significant dilemma for dental practitioners due to the disease’s complexity and severity. Various approaches and materials have been employed to address this condition through both surgical and nonsurgical interventions; among these, HA has demonstrated favourable outcomes in both treatment modalities [80]. HA, a non-sulfated glycosaminoglycan, exhibits hygroscopic properties, allowing it to increase its weight by more than 50 times through water absorption. This characteristic contributes to the ECM’s viscoelasticity. It enhances tissue hydration, facilitating the exchange of gases and small molecules, while simultaneously acting as a barrier against viruses, bacteria, and macromolecules [80]. Furthermore, HA possesses bacteriostatic, anti-inflammatory, non-antigenic, and antioxidant properties [81, 82]. Collectively, these attributes confer an antibacterial effect that inhibits both bacterial adhesion and biofilm formation, even under prolonged application. The antibacterial efficacy of HA is also influenced by its molecular weight. Low-molecular-weight (LMW)-HA (<300 kD) encourages cell differentiation and proliferation while exhibiting inflammation-suppressing effects. Conversely, there is growing evidence that high-molecular-weight (HMW)-HA (1,000 kD) may exert an immunosuppressive influence, potentially mitigating excessive inflammation [83].

A clinical trial (randomised control) was conducted to assess the influence of HMA-HA gel on the subgingival microbiomes associated with implants exhibiting peri-implantitis after a minimum period of 1 year of loading. The 16S rRNA sequencing technique was utilised to analyse the impact of HA gel on the subgingival microbiomes. The data revealed that the application of HA gel in the test group significantly decreased microbial abundance compared to the control group, in which microbial proliferation increased. Furthermore, the results demonstrated that HA application produced a greater reduction in the presence of microorganisms associated with the early colonisation of peri-implantitis (specifically *Streptococcu*s, *Veillonella*, and *Rothia*), in addition to a moderate effect against middle colonisers (*Prevotella* and *Campylobacter*). However, HA was found to be ineffective against later colonisers, suggesting that the action of HA is insufficient once peri-implantitis has progressed to include late colonisers. Notably, HA gel did not influence the non-oral bacterial species (*Ralstonia* and *Sphingomonas*). This study indicates the HMW-HA’s potential protective effect against peri-implant complications by limiting bacterial colonisation and growth [84].

In a related randomised controlled trial (RCT), Fernández et al. [41] also assessed the progress of peri-implantitis and peri-implant pocket after treating it with the topically applied HMW-HA by evaluating the crevicular concentration level of pro-inflammatory mediators (interleukin [IL] 1β and tumour necrosis factor [TNF]) along with other clinical variables (BOP, PD, CAL [clinical attachment loss], and peri-implant bone loss) at three timelines, which are: baseline (0), 45, and 90 days. The findings demonstrated that the test group, in which peri-implant areas were treated with topical HA, exhibited the most significant reduction in bleeding at both the 45- and 90-day time points. PD, attachment levels, and bone loss were markedly decreased in the test group compared with the control group, with the differences particularly pronounced at the 90-day evaluation, as detailed in Table 3. Enzyme-linked immunosorbent assay (ELISA) analysis, which evaluated the crevicular concentrations of cytokines IL-1β and TNF, indicated that the test group had lower concentrations of inflammatory cytokines than the control. However, these differences were primarily observed in cases where PD exceeded 5 mm, suggesting that HA application had a more pronounced effect against progressing and advancing lesions. This study corroborates the therapeutic efficacy of topical HMW-HA as a non-surgical management strategy for peri-implantitis [41].

**Table 3:** Result of clinical variables evaluated after topical application of HMW-HA [41].

Variable	Baseline	45 days	90 days
**BOP (%)**	100 (vs. 100, 100)	15.6 (vs. 28.1, 33.3)	15.6 (vs. 28.1, 38.9)
**Peri-implant pocket depth (mm)**	3.62 ± 0.83 (vs. 4.21 ± 1.14, 3.67 ± 0.72)	3.08 ± 0.85 (vs. 3.79 ± 1.00, 3.35 ± 0.72)	2.97 ± 0.64 (vs. 3.63 ± 1.01, 3.25 ± 0.78)
**CAL (mm)**	4.06 ± 1.40 (vs. 4.37 ± 1.32, 3.74 ± 0.76)	3.52 ± 1.45 (vs. 3.95 ± 1.17, 3.41 ± 0.74)	3.45 ± 1.49 (vs. 3.79 ± 1.24, 3.29 ± 0.75)
**Peri-implant bone loss (mm)**	3.41 ± 1.66 (vs. 3.56 ± 2.01, 3.40 ± 0.91)	3.40 ± 1.63 (vs. 3.65 ± 1.99, 3.41 ± 0.87)	3.39 ± 1.70 (vs. 3.66 ± 2.04, 3.43 ± 0.89)

BOP: bleeding on probing; CAL: .

Note: Values in parentheses indicate results for Control 1 and Control 2, respectively.

Zhoe et al. [85] conducted an *in vitro* and *in vivo* study, developing a novel hybrid gel composed of HA and chitosan, loaded with the anti-inflammatory drug dexamethasone (HA-CT-DE) for the repair of peri-implantitis. *In vitro* characterisation of this hydrogel demonstrated its enhanced biocompatibility. *In vivo* analysis evaluated the degradation of the hydrogel and repair of peri-implantitis in mice, and the results showed that the HA-CT-DE hydrogel demonstrated sustained release, an equilibrium swelling of 18, and the ability to stimulate growth against NIH-3T3 fibroblast cells. The expression levels of TNF-α, IL-6, and IL-1β, three inflammatory factors, were down-regulated in peri-implantitis. HA-CT-DE hydrogels have been demonstrated *in vitro* to inhibit methicillin-resistant *Escherichia coli* and *Staphylococcus aureus* [85].

**Figure 5:**
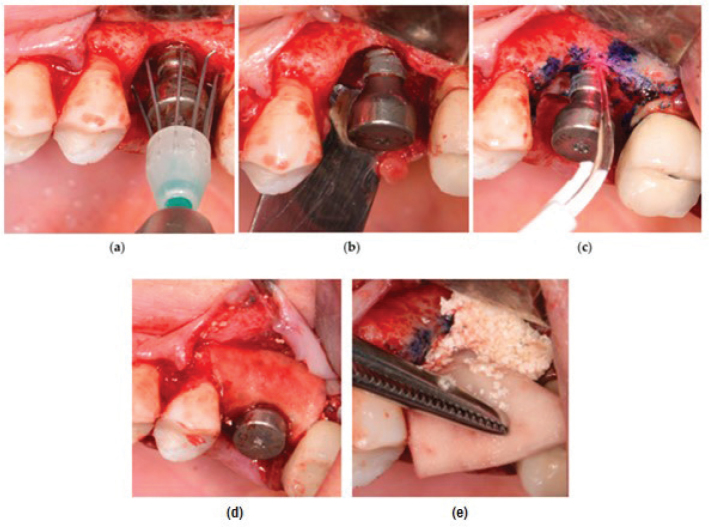
The stepwise successful application of bovine bone combined with HA at peri-implantitis sites involves the following sequential procedures: (a) mechanical debridement of the defective site, (b) assessment of the defect after mechanical debridement, (c) disinfection of the surfaces of the implant utilising photodynamic therapy, (d) application of bovine bone with HA, and (e) coverage of the bony graft with a porcine dermal collagen matrix [86].

**Figure 6:**
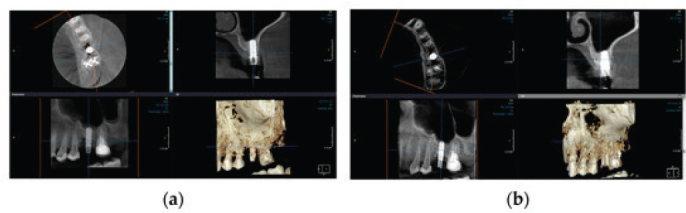
Shows the (a) bone level at baseline before the surgical management of peri-implantitis and (b) after 6 months of surgical intervention displaying significant bone gain [86].

**Figure 7:**
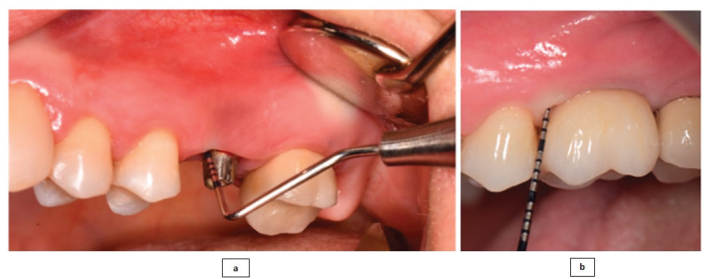
Shows (a) increased probing depth before the surgical management of peri-implantitis with HA and (b) significantly improved probing depth after 6 months of surgery with bovine bone merged with HA [86].

Recent research has also employed HA in the surgical treatment of bony defects resulting from the destruction caused by progressive peri-implantitis. A pilot study carried out by Rakaševi et al. [86] evaluated the effectiveness of a combination of a bovine bone substitute and HA in addressing bony defects associated with peri-implantitis. The study explored its effectiveness through radiographic analysis and various clinical parameters. The HA-combined bovine bone was successfully placed at the peri-implantitis-induced bony defect site (Figure 5). The radiographical findings, as shown in Figure 6 indicated that the HA-treated test groups exhibited greater vertical marginal bone gain and improved implant stability quotient after 6 months in comparison to the control group, which only received the bovine bone substitute. Clinically, an improved clinical outcome, as shown in Figure 7, with no BOP was observed, and the pocket depth also decreased to less than 5 mm, suggesting the potential of HA as a promising agent in the surgical reconstructive treatment of peri-implantitis [86].

Valverde et al. [87] conducted a systematic review and meta-analysis to evaluate the effectiveness of HA in treating peri-implantitis. The results of the study concluded that HA alone or in combination with other materials was effective for the treatment of peri-implantitis, as the meta-analysis demonstrated a highly significant difference favouring the HA-treated group over the control group (*p* = 0.00001). However, considerable heterogeneity was found between studies (*I* ^2^ = 93%) due to differences in their study design, surgical and non-surgical treatment modalities, dosage, and outcome assessment. Therefore, the researcher recommended carefully planned RCTs with extended follow-up times that demonstrate the advantages of HA in the management of peri-implantitis are required and justified [87].

Table 4 summarises studies that have used HA for the therapeutic management of peri-implantitis and its associated defects.

**Table 4:** Studies utilising HA for the non-surgical and surgical intervention of peri-implantitis.

Study design	Objective of the studies	Material used	Results	References
Experimental study (*in vitro* and *in vivo*).	Synthesis of HA and chitosan composite loaded with dexamethasone. Evaluation of physicomechanical characteristics and biocompatibility of the hydrogel through *in vitro* testing. Hydrogel disintegration and peri-implantitis healing were investigated *in vivo*.	HA and chitosan composite loaded with dexamethasone.	*In vitro*: The hydrogel had adequate gelling time and viscosity with a swelling equilibrium of 18. It also reduced the expression of pro-inflammatory cytokines and showed and displayed marked antibacterial effect against methicillin-resistant species of *S. Aureus* and *E. Coli.* *In vivo*: Controlled degradation of hydrogel was observed and it was resorbed entirely between 4 and 6 weeks. Reduction of inflammation and new tissue regeneration were noted at the implant site.	[85]
Prospective case series.	Evaluation of the effectiveness of ribose cross-linked collagen matrices with functionalised cross-linked HA for treating the defects associated with advancing peri-implantitis through clinical and radiographic parameter.	Ribose cross-linked collagen matrices with functionalised cross-linked HA.	Clinical findings: Bleeding on probing frequency dropped from 63% to 10%, and an average 3.9 ± 1.85 decrease in probing depth was noted after 12 months. Radiographic findings: 1.02 mm gain in marginal bone level was achieved around the treated implants.	[88]
*In vitro* study.	Evaluation of the influence of HA, chlorhexidine, and azithromycin on the expressions of the genes (fimA, mfa1, hagA, rgpA, rgpB, and kgp), associated with the *P. gingivalis* adhesion and virulence, the most common cause of peri-implant tissue damage.	HA, azithromycin, and chlorhexidine.	HA had a significant effect on all the genes except rgpB, and it reduced the expressions of all the genes by almost half-fold, with fimA, mfa1, hagA, rgpA, and rgpB, and kgp genes showing 0.35 0.20, 0.47 0.35, 0.44 0.25, 0.67 0.46, 0.48 0.33, and 0.35 0.22 values. HA with lower concentrations presented higher gene down-regulation, and 1 mg/mL was found to be highly effective. HA had a lower overall effect than AZM but a higher impact than CHX.	[89]
Pilot study –Non-randomised clinical trial.	Evaluation of the effectiveness of the nebulised HA for managing peri-implantitis.	Nebulised HA.	A slight improvement in probing depth was noted after 15 days at HA-treated sites; however, the difference between control and test sites was not statistically significant. Bleeding on probing was equally improved in the test and control sites.	[90]

HA: hyaluronic acid.

### HA in implant surface modification

Implant coatings should be used to improve osseointegration, a crucial component of dental implant success [91]. The surface characteristics of implant coatings play a significant role in osseointegration and bone healing [91]. Currently, the coating of implants has been enhanced using various natural polymers and organic materials. One method also involves modifying the implant surface with extracellular organic matrix [92]. HA renders the coating surface hydrophilic, thereby attracting various growth factors and proteins that facilitate bone healing mechanisms and osteointegration during implantation [93]. As a coating material, HA can adhere covalently to the metallic surfaces of implants [94].

HA has the potential to accelerate bone regeneration through mechanisms including proliferation, chemotaxis, and mesenchymal cell differentiation. It is also associated with osteopontin and bone morphogenetic protein-2 [95]. A study has revealed a synergistic effect on bone density, both in quality and quantity, exhibited by HA, which contributes to the stability of the implant during placement in the posterior maxilla. This stability is contingent upon the duration of placement and the healing process [14]. Hamdy et al. conducted a randomised clinical trial [96] to evaluate and compare the implant stability of HA-coated dental implants with SLA dental implants (Sandblasted Large Thread Acid Etched) at 7 and 14 days after implant insertion in the posterior maxilla. Resonance Frequency Analysis (RFA) was utilised to evaluate implant stability, while the Landry index assessed soft tissue healing (surgical wound healing). The statistical analysis showed that the two groups’ patterns of implant stability differed. Across all time points, implant stability in the SAE implant group declined considerably from insertion to the 12th week (*P* ≤ 0.001). On the other hand, from insertion to 12 weeks, the stability of the HA-coated implant group improved significantly overall (*P* = 0.011). Between weeks 6 and 12, stability increased significantly (*P* ≤ 0.001) despite a non-significant decrease between insertion and 6 weeks (*P* = 0.244). HA-coated dental implants also demonstrated significantly higher soft tissue healing (*p* = 0.005) than SLA dental implants at all time points [96].

Another *in vitro* study [97] evaluated the potential functionality of HA-coated polyether ether ketone (PEEK) implants by assessing the viability, differentiation, growth, and osteogenic activity of MG-63 human osteoblastic cells. The results indicated that HA-coated PEEK enhanced cell viability, proliferation, ALP activity, and mineralisation potential, indicating the ability of HA to improve the biofunctionality of dental implants [97].

In addition, HA can provide a bacterial-resistant coating for implant surfaces [98]. HA biopolymer has emerged as a promising material for the creation of biocompatible, antibacterial coatings on dental implants. However, the sole bacterial-repelling mechanism provided by conventional HA-based coatings might not be sufficient for long-term protection [99]. Recent studies have focused on developing multifunctional or bifunctional antibacterial coatings that integrate synergistic mechanisms to enhance efficacy [78]. Hydrogel-based HA coatings could improve the antibacterial properties of Ti6Al4V implants by preventing microbial penetration and biofouling, owing to their crosslinked three-dimensional hydrophilic network with high hydration capacity [100]. Moreover, although HA alone lacks intrinsic bactericidal activity [101], HA-based hydrogel coatings can be engineered to provide sustained release of antimicrobial agents [102].

An *in vitro* experimental study done in 2023 [103] evaluated the sustained drug release ability and antibacterial activity against two most commonly involved pathogens of HA-based hydrogel coating on Ti6Al4V implants involved in implant-associated infections: Staphylococcus aureus (S. aureus) and *Escherichia coli* (*E. Coli*). Compared with uncoated Ti6Al4V surfaces, HA-based hydrogel coatings exhibited a considerable decrease in SYTO-9 fluorescence intensity for both S. aureus and *E. coli*, suggesting effective bacterial-repelling capabilities. Surprisingly, these coatings also demonstrated a novel bacterial-killing mechanism that killed both bacterial strains, a property never observed in HA-based materials. This antibacterial activity is believed to result from the hydrogel’s high swelling capacity, which makes it easier for bacteria to absorb and become trapped within its microporous network, thereby restricting their survival. HA-based hydrogel coatings thus exhibit a more potent bactericidal mechanism against *E. coli* and a greater resistance to S. aureus [103].

Another experimental study used a selenium and HA coating (Se/HA) on CP-Ti (commercially pure titanium) dental implants and assessed its antimicrobial potential against S. aureus and *E. coli* by measuring zones of inhibition. Dip coating was used to apply a Se/HA coating to the implant. The Se/HA coating showed significantly higher antibacterial activity against both pathogens, particularly against S. aureus, compared to the control [98]. Table 5 lists studies that have used HA-based coatings to modify implant surfaces.

**Table 5:** The role of HA and its impact on dental implants.

Coating	Results	Reference
VEGF on Ti surface functionalised through a grafted layer of a combination of carboxy-methyl chitosan (CMCS) with hyaluronic acid-catechol (HAC).	HA inhibited bacterial growth and promoted osteoblast functions. Calcium deposition and mineralisation improved on the titanium surface.	[104]
Dental implants made up of Ti and coated by an ECM that was artificially formed, consisting of collagen type I and low sulfated hyaluronan (sHA) derivatives	Peri-implant area formation of bone was increased in the maxillary bone around the implant. No signs of inflammation was observed. sHA1 increased the activity of osteoblast.	[92]
HA formed hydrogel coatings on Ti6Al4V implants.	Excellent biocompatibility response was observed along with antibacterial functions such as repelling and killing bacteria. This coating also stimulated cellular-based activities such as proliferation activity, differentiation and mineralisation.	[103]
HA/CS multilayers on Ti6Al4V implants.	Reported antibacterial activity against S. aureus. This study reported the smooth pattern of multi-layer coatings act as reservoir that serve the function of bactericidal activity around the implant.	[105]
Ti Surface with PTL to form multilayer coating composed of Ag/CS/HA.	AgNP destroyed the bacteria until healing is achieved. However, showed some cytotoxicity that can be controlled by Ag concentration.	[106]
HA gel around the implants.	New bone and osteoid tissues formed at the implant site. Healing of bone also occurred.	[12]
The surface of Ti implants treated with sandblast, machine, sandblast plus acid-etched, HA, (HAp).	HA-coated surface showed better osseointegration than other treated surfaces.	[107]
Biphasic calcium phosphate (BCP) with HA.	New bone formation and healing of bone occurred. Osteoinductive properties also improved.	[108]
HA Gel with Simvastatin around the dental implants.	Osseointegration was improved. Stability of implant achieved along with an increase in density of bone.	[109]
Coating of HA bisphosphonates on PEO-based metal implants.	Decreased the bacterial adhesion and coating used was also non-toxic.	[76]
HA and flowable L-PRF coatings.	HA provided more stability than flowable L-PRF. Improved osseointegration.	[110]
Coating of HA and selenium on the implant surface.	Enhanced resistance to corrosion. Good activity of antimicrobial found against S. aureus.	[98]
Polyether ether ketone coated with HA.	Survival of proliferation and differentiation of cells was enhanced. Improvement was found in mineralisation.	[97]

PRF (Platelet-rich fibrin); BMP (bone morphogenetic proteins); ECM (extracellular matrix); LXR (liver X receptors); SAE (sandblasted acid etch); CBCT (Cone Beam Computed Tomography); CAL (Clinical attachment loss); ECM (extracellular matrix); PTL (phase-transited lysozyme).

### HA in soft tissue healing around implants (peri-implant seal)

The success of implant therapy is contingent not only upon the efficient osseointegration of the implant with the alveolar bone but also on the attachment of adjacent soft tissue (epithelial and connective tissue) to the implant [111]. This soft tissue-implant interface functions as a biological seal that separates the underlying bone and implant from the external environment of the oral cavity through the implant adhesion with the epithelial and connective tissues, thereby inhibiting the build-up of bacterial plaque in the implant sulcus (which is formed between the implant and associated soft tissues); thus inhibits the onset of peri-implant inflammatory diseases [111]. Consequently, the proper healing of soft tissue around the implants serves an essential part in the functionality and durability of dental implants by creating a biological peri-implant seal.

The biological process of soft tissue healing is dynamic and intricate, encompassing key stages such as fibroblast differentiation and growth, ECM deposition, re-epithelialisation, and angiogenesis (new blood vessels formation) [112–114]. Therefore, the repair and healing of soft tissues rely heavily on the extent of collagen matrix deposition, vascularisation, and the availability of amino acids required to support increased metabolic activity at the healing site. In clinical practice, shorter healing times are essential, as they enhance patient outcomes by reducing post-operative pain and swelling, ensuring adequate haemostasis, and lowering the risk of infection [115–117]. Therefore, new topical medications containing chemicals or compounds that facilitate repair have been developed to enhance healing and repair of soft tissues around the implants. One such advancement is the availability of commercial topical gels (HAplus gel and Aminogam gel) consisting of a combination of HA (LMW) and collagen-producing amino acid precursors (proline, leucine, lysine, and glycine), aimed at improving the healing of the mucosal epithelium and connective tissue [118–120]. HA is widely used for its ability to form transient structures that facilitate the assembly and deposition of extracellular matrix proteins, promote cell attachment, migration, and proliferation, and modulate vascular endothelial cell activity [121–123]. Furthermore, HA supports tissue hydration and ECM resilience [122]. Previous studies have also indicated that HA-based gels promote faster healing of oral soft tissue post-surgery and reduce postoperative complications. Aminogam gel, a HA-based gel, contains not only HA but also four essential amino acids (glycine, leucine, proline, and lysine), which are required for wound healing. Glycine stimulates fibroblast proliferation and collagen deposition, lysine mediates the inflammatory response by modulating cytokine expression, and leucine promotes angiogenesis and proline to fasten wound healing by increasing the proliferation and migration of keratocytes. All these components give aminogam the ability to heal wounds and soft tissues, reduce pain, and exhibit anti-inflammatory and anti-odematous properties [120, 124].

**Figure 8:**
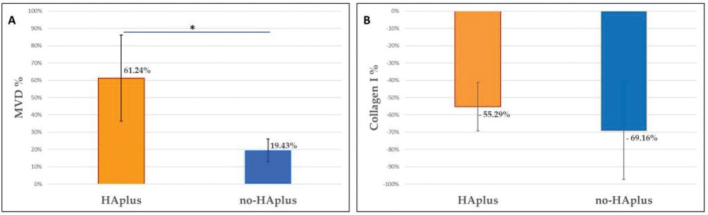
Shows that (A) the HA plus (amino acids –enriched HA gel) treated group resulted in higher neo-vascularisation than the non-HA treated group and (B) a non-significant difference was observed between the collagen content of the treated and non-HA treated group [125].

Canciani et al. [125], in their experimental research (*in vitro* and *in vivo* studies), evaluated the influence of an amino acid-enriched HA gel, with and without the incorporation of vitamins C and E, on the healing of the oral mucosa. The findings indicated that the application of the gel to the treated site resulted in increased neo-vascularisation, as depicted in (Figure 8), and improved organisation of collagen fibres. However, the difference in collagen concentration between the HA-treated and non-treated sites was not statistically significant (Figure 8). Nonetheless, the HA gel reinforced with vitamins C and E demonstrated a greater potential to enhance collagen production during the healing process, as evidenced by its positive stimulatory effect on fibroblast cells in culture. A retrospective study conducted in 2020 [126], evaluated the healing time of soft tissue after endosseous implant placement following the application of aminogam gel on the peri-implant soft tissues. The results indicated that the healing time in the experimental groups (treated with topical application of aminogam gel) was reduced by 28% (6 days less than the control group), with complete healing of the mucosa and no surgical complications. Histological examination also revealed that the aminogam gel led to the development of well-differentiated and developed connective tissue with lower concentrations of inflammatory infiltrate and vascular density [126]. These studies indicate that amino acid-enriched HA gel can significantly improve the peri-implant seal by positively influencing soft tissue healing around the implant, thereby optimising clinical and patient outcomes.

Bawankar et al. [127] conducted a RCT in 2024 to evaluate the effectiveness of injectable HA with microneedling (MN) against microneedling alone for enhancing peri-implant soft tissue volume. RCT consisted of two groups: Group 2 received HA on the peri-implant mucosa after MN, while Group 1 received only MN. At baseline, 3, and 6 months, clinical measures, including the Plaque Index, Gingival Index, PD, attachment levels, bleeding index, mucosal thickness, keratinised tissue width, interproximal width, and papillary recession height, were measured. HA combined with MN led to increased mucosal thickness and keratinised tissue width, along with a reduction in peri-implant inflammation and greater soft tissue regeneration, indicating an overall gain in soft tissue volume. This shows that HA can be a promising material for enhancing soft tissue around implants [127].

### HA as a scaffold in regenerative tissue engineering

A sufficient amount of alveolar bone is necessary for dental implant placement. If the bone quantity is insufficient, bone replacement can be achieved through grafting or regeneration procedures. Conventional grafting methods, such as autografts, allografts, and xenografts, which have been widely used for alveolar bone regeneration, also have notable limitations. Autografts require a second surgical site and have limited availability. In contrast, allografts and xenografts do not require harvesting but pose risks of immune reactions, inconsistent integration, delayed remodelling, and potential disease transmission [128]. Modern dentistry is now shifting towards bone regeneration through tissue engineering, an advanced approach that combines scaffolds, cells, and [129] signalling molecules to replicate the natural bone environment for targeted bone formation [130].

For bone tissue engineering (bone regeneration through tissue engineering), three essential elements are necessary for effective bone formation: (1) osteoprogenitor cells that can differentiate into functional bone cells, such as mesenchymal stem cells (MSCs), induced pluripotent stem cells (iPSCs), or embryonic stem cells (ESCs); (2) specific growth factors that promote cell migration, proliferation, differentiation, and formation of blood vessels; and (3) a scaffold that serves as a 3-dimensional structured framework for osteogenic cells to adhere and grow. Among these three key elements, the scaffold plays a crucial role in creating a suitable environment for osteogenic stem cells to adhere and grow with the presence of growth factors necessary for new bone formation. A scaffold for bone regeneration should ideally possess several key characteristics. It must be biocompatible, osteoinductive, antimicrobial, and anti-inflammatory, while also promoting tissue growth and vascularisation. In addition, the scaffold should be biodegradable, with a degradation rate that supports new bone formation. It must provide adequate structural and mechanical support for the defective area and feature a porous structure to facilitate healing [129]. Thus, the selection and preference of scaffold material constitute a critical step in bone tissue engineering [131].

Various materials have been utilised for scaffold fabrication, among which HA has been used due to its promising properties [132, 133]. As a scaffold for bone regeneration, HA offers significant advantages. It is biocompatible, biodegradable, antioxidant, anti-inflammatory, and found naturally in connective tissues, where it promotes cell proliferation, differentiation, and lubrication – properties that can be transferred to engineered scaffolds. HA has functional groups that allow it to be chemically modified or cross-linked to create hydrogels, and it can create a highly hydrophilic environment that supports cell infiltration and growth. Furthermore, HA is bioactive in both its intact and degraded forms, maintaining its functionality throughout the regenerative procedure [134]. Hydrogel HA scaffolds, a three-dimensional network of HA molecules that are crosslinked to form a gel-like material with high water content, are mostly utilised for bone tissue regeneration as hydrogel-based HA scaffolds exhibit optimal mechanical properties, enhanced biocompatibility, increased flexibility, manageable network architecture, and the ability to resist dissolution in water [133, 135]. HA hydrogels have a high water content, which makes them highly permeable to nutrients, oxygen, and metabolites [136]. Sponge and meshed forms of HA scaffolds are also used in tissue engineering, as they can be modified chemically and structurally to meet the essential requirements of bone regeneration [137].

Several studies have used HA-based scaffolds to regenerate alveolar or maxillofacial bone, yielding promising results. A 2022 critical review by D’Albis [133] analysed results from various studies that used HA as a scaffold for alveolar bone regeneration. This study reviewed eight studies, and the combined findings indicate that HA can be used as a scaffold in combination with other biomaterials to regenerate alveolar bone due to its promising osteogenic and osteoinductive properties [133]. A study conducted by Hamlet et al. in 2017 [138] evaluated the potential of mPCL (medical grade polycaprolactone) containing osteoblasts embedded in an HA-based scaffold-rich in bone morphogenetic protein-7 (BMP-7) for alveolar bone regeneration through *in vitro* and *in vivo* testing. The *in vitro* findings demonstrated that osteoblasts encapsulated in HA-hydrogels remained viable and generated a mineralised collagenous matrix over 6 weeks, thereby confirming effective differentiation. *In vivo*, mPCL–hydrogel constructs exhibited a significantly larger volume of vascularised bone-like tissue after 4 weeks, indicating improved osteogenic performance [138]. An *in vivo* study also investigated the potential of a collegena-encapsulated hydroxyapatite (HA) and calcium sulphate (CS) composite (HAP/CS/HA-Col) for alveolar bone regeneration in rats. The HAP/CS/HA-Col composite exhibited the highest new mature bone formation, with mechanical properties similar to those of natural trabecular bone and excellent biocompatibility [139]. Another study fabricated the novel dental plug composed of HA, tri-calcium phosphate, and biphasic calcium phosphate for single-step bone grafting in the extraction socket and evaluated its properties. The findings revealed that the designed HA-based dental plug was biocompatible and osteoconductive with excellent haemostatic properties. This HA-based scaffold, when tested *in vitro*, showed greater and improved bone formation in a rabbit femur model [58]. A hydrogel scaffold based on HA, chondroitin-6-sulfate, and deratan sulfate, and high-molecular-weight heparin, has demonstrated favourable osteogenic-promoting properties *in vivo* [140].

Azar et al. [141] explored the latest developments in the tissue engineering triad for dental implant therapy using a HA scaffold in combination with oxysterol and human dental pulp stem cells (hDPSCs) in their study. Human dental pulp stem cells have vascular endothelial growth factors and fibroblast growth factors and produce osteoblast during the bone repair process. They also upregulate gene expression of osteopontin, alkaline phosphatase, and osteocalcin, thereby increasing vascularity and accelerating bone formation. Oxysterol, an oxidised form of cholesterol, which is found naturally in the human body, promotes osteoinduction by activating LXRs and the Hedgehog (Hh) signaling pathway

liver X receptors (LXRs) (Hh) signalling pathway through binding with Smo (smoothened receptor). Hence, it promotes bone development. The scaffold HA base is used to deliver various mediators or osteoinductive substances. The scaffold’s structure is maintained in a hydrated environment by HA distributed throughout it, made possible by its viscosity and the chelation between hyaluronate and Ca^2+^. The study concluded that the combination of oxysterol and hDPSCs grown on HA scaffolds may act as an osteoinductive substance to accelerate the bone repair and regeneration process during dental implant treatment [141]. 3-D printing technology is also increasingly being used to produce HA-based scaffolds with greater accuracy and customisation. An *in vivo* study evaluated the osteogenic potential of 3-D printed polylactic acid (PLA) and HA-based scaffolds, compared with a control (no graft) and a 3-D printed PLA group, in a rabbit calvaria model. The results indicated that the 3-D printed scaffold made of PLA and HA displayed the highest bone-to-tissue volume ratio, showed no adverse reaction, and had improved integration with the margins of the bony defect [136]. Another study fabricated a composite scaffold via 3-D printing for bone regeneration. This composite was made by combining HA, gelatine, and hydroxyapatite. The study reported improved cellular proliferation and osteogenic differentiation potential [115].

However, HA scaffolds exhibit a significant limitation relating to biodegradation. During the degradation process, they modulate the inflammatory response, leading to the release and elimination of low-molecular-weight compounds. Consequently, this suppresses leukocyte migration and neutrophil adhesion [116]. The disadvantages of HA scaffolds, including degradation and occasional poor mechanical strength, can be mitigated through crosslinking, chemical modifications, alterations in pore size and volume, and the incorporation of rigid additives such as bioceramics and bio-glass [117, 142].

**Figure 9:**
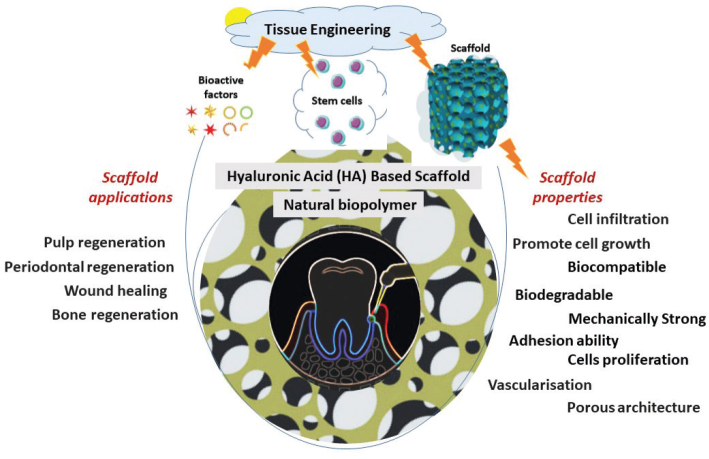
Representation of the wide applications and properties of HA-based scaffolds in tissue engineering.

Figure 9 summarises the characteristics of scaffolds, while Table 6 provides an overview of recent studies on HA scaffolds for dental applications.

**Table 6:** Applications of HA scaffolds in dentistry.

S.NO.	Scaffold	Results	Reference
1.	Methacrylated HA microwell scaffold.	Inhibition of bacteria by HA and promotion of osteoblast functions. Calcium deposition and mineralisation improved on titanium surface. Two cells were studied in this study ‘human adult low calcium high temperature (HaCaT) cells’ and ‘dental pulp stem cells (DPSCs)’.	[143]
2.	Recombinant human bone morphogenetic protein with HA represented as (rhBMP-2 HA hydrogel).	Formation of new bone. Osseointegration improved.	[144]
3.	Click-crosslinking with HA hydrogel scaffold morphogenetic protein-2 (BMP-2) mimetic peptide (BP).	Good biocompatibility. Increased osteogenic differentiation. Better compressive strength. Human dental pulp stem cells (hDPSCs) were studied in this study Growth factors such as BP or BMP-2 (mimetic peptide) and (bone morphogenetic protein-2) respectively were used.	[145]
4.	Coating of HA hydrogel on the surface of Ti6Al4V implants.	Excellent biocompatibility. Improve bactericidal activity. “Human dental pulp stem cells (hDPSC)’ were studied in this study.	[103]
5.	Hybrid based on CS-HA hydrogel scaffold (periodontal regeneration).	Viability of cells was increased. An increase in cell migration was reported. Mouse fibroblast cell line (NIH-3T3) and human osteoblastic line (MG-63).	[146]
6.	HA-based hydrogel (Restylane) as an injectable type scaffold (pulp regeneration).	This injectable hydrogel effectively promoted the viability of cells and mineralisation. Also, the differentiation of odontoblast-like cells also promoted. ‘Stem cells of the apical papilla (SCAP)’ were studied.	[147]
7.	HA-based scaffold with a coating of bilayer cellulose (wound healing inside the oral cavity).	Cytocompatible. Improved recruitment of fibroblast cells as well as proliferation. Increased healing observed.	[148]
8.	HA with extract of Cissus quadrangularis-based scaffold for (periodontal regeneration).	Tenogenesis potential enhanced. Tensile strength and orientation of collagen fibres increased. ‘Mesenchymal stem cells’ were studied in this study.	[149]
9.	Sponge of collagen with HA-based scaffold (periodontitis).	This scaffold showed no evidence of adverse reaction. Wound healing improved.	[150]
10.	HA in salivary gland regeneration.	High molecular weight HA increased the proliferation and organisation of c-Kit+ progenitor cells. The germ formation of salivary gland organs was enhanced.	[151]

HA: hyaluronic acid.

### Complications of HA and their management

To enhance the regenerative properties and handling characteristics of bone, HA with different molecular weights are utilised. Sieger et al. [152] investigated the tissue response and inflammatory biocompatibility of two molecular weights of HA in combination with biphasic bone grafts. Both *in vitro* and *in vivo* tests were performed in the study. L929 cells were used for the analysis of HMW and LMW HA cytotoxicity. The initiation of anti- and pro-inflammatory macrophages, as well as multinucleated giant cells (BMGCs), was assessed using histological, immunohistochemical, and histomorphometric methods across these three materials. The researchers found that all materials were nontoxic and did not cause biological damage to L929 cells. For *in vivo* evaluation, calvarial defects were created in 20 Wistar rats, which were subsequently filled with a biphasic bone substitute (BBS) or with BBS combined with two different HA dosages. A marked declination in pro-inflammatory macrophages was observed in a group where a high dose of HA was incorporated into the BBS, as compared to the control. M2 macrophages and BMGCs were not detected in any of the study groups, yielding statistically nonsignificant results. M2 macrophages are activated by exposure to specific cytokines, such as IL-4, IL-10, and IL-13, and are responsible for wound healing and tissue repair by enhancing collagen synthesis, producing either polyamines or proline [153]. Empty defects were used as the study’s control group. The authors indicated that the inflammatory response to BBSs was not affected by the addition of HA.

To address the issue of missing interdental papillae, Bertl et al. [154] utilised mucosal HA (HY) gel injections around implant-supported crowns. They evaluated two patients with single non-neighbouring implants in the upper arch. The injections were administered using a three-step technique bilaterally, with a protocol that included a repeat injection session approximately 4 weeks later. The researchers noted that after the second injection, puffiness, marked pain, and a tingling sensation in the lip adjacent to the injection site were observed. In one patient, livedo reticularis, a lattice-like altered skin colour change was evident; however, it was documented that these symptoms resolved after 7 days without resulting in necrosis or permanent skin damage.

Product migration, delayed swelling, and inflammatory reactions were noted when resorbable tissue fillers containing HA were employed. The occurrence of these inflammatory reactions is hypothesised to be occurring because of the cross-linking of HA fillers. De Jong et al. [155] developed five experimental HA fillers with progressing levels of cross-linking agents. In cytotoxicity evaluations, absent to minimal cytokine production was observed when human-derived macrophages (THP-1) were exposed to the HA fillers. In addition, gene expression analysis indicated changes in the control of cell cycle and immune functions. The most prevalent complications associated with HA include intravascular necrosis, skin necrosis, biofilm-related infections, and foreign body granuloma.

#### Intravascular necrosis

Intravascular necrosis leading to embolia cutis medicamentosa following the administration of HA is a common complication [156, 157]. Full-thickness necrosis, eschar, and scarring may occur within a timeframe ranging from 48 h to 7 days post-application of HA [158]. Intravascular necrosis may result from the use of sharp needles, deep injections, and the administration of large boluses at a single site. Common complications include intense pain, ischemia, webbed-like skin pattern, and purple-red mottling attributable to blood-filled blisters. To mitigate the effects of HA-related complications, hyaluronidase should be administered as promptly as possible [158].

#### Skin necrosis

The most serious complication associated with HA is skin necrosis, which results in anaesthetic scar alterations. It has been posited that this phenomenon may be attributed to both extravascular and intravascular factors; however, the precise mechanism remains incompletely understood. Extravascular factors include reduced skin perfusion when excessive HA is introduced, which can lead to external vascular compression [159]. The prominent symptoms of skin necrosis include tissue reactions, such as oedema and inflammation. Allergic reactions and inflammation may occur when HA contains trace amounts of foreign proteins [160]. In terms of intravascular factors, high-molecular-weight HA can obstruct arteries and induce chemical damage to the endothelial lining [161]. For HA vascular complications, there is currently no standardised treatment protocol. Treatment options include hyaluronidase injection, prostaglandin E1, nitroglycerin paste, and warm compression and massage aimed at disrupting the filler embolus. However, it is important to note that there is still a scarcity of data that claims or proves that skin necrosis resulting from vascular complications can be reduced with hyaluronidase, despite its effectiveness in correcting unfavourable outcomes associated with HA within 24 h [162, 163].

#### Biofilm causing infection

The most prevalent human bacterial colonisers of skin and mucous membranes are members of the genus *Staphylococcus*, with *Staphylococcus epidermidis* being the most frequently isolated species from human epithelial tissues [163]. *Staphylococcus epidermidis* is associated with a range of infectious processes, including urinary tract infections, ear infections, respiratory tract (upper) infections, dental plaque formation, gingivitis, and prosthesis-associated infections. In certain circumstances, the infection can progress to endocarditis [163, 164]. These bacteria exhibit a propensity to adhere to implant surfaces by secreting an adhesive and protective matrix of proteins that facilitate further accumulation and attachment of microorganisms on the implant surface. Furthermore, infections can arise from biofilms on needles used to inject HA [163, 165]. Rarely, weeks after these injections, patients may present with persistent, erythematous, and tender nodules. Abscesses or sinus tracts may develop as a consequence of these infections. In most cases, these infections persist for extended periods, often being misdiagnosed and inadequately treated as allergic reactions, as the bacteria are frequently culture-negative [165]. Broad-spectrum antibiotics can effectively address these infections; however, it is critical to avoid immunosuppressive steroids, as they may exacerbate the condition [166].

#### Foreign body granuloma

Following the administration of HA, granuloma formation occurs within 5 to 15 months, characterised by the presence of histiocytic and multinucleated giant cells, collagen deposition, and phagocytosis in the surrounding connective tissue. Foreign body granulomas present clinically as substantial facial swelling and erythema, accompanied by one or more nodules. While most of these granulomas are asymptomatic, they can be aesthetically distressing to patients. In more severe cases, slight irritation, localised tenderness, swelling, vascular lesions, redness, and ulceration may be observed. Pathologies, including orofacial granulomatosis, erysipelas, contact dermatitis, facial oedema, and sarcoidosis, must be considered as differential diagnoses during patient history and examination [167]. Treatment options for this condition include antihistamines, systemic corticosteroids, topical tacrolimus, minocycline, retinoids, allopurinol, 5% imiquimod, and surgical excision [167, 168].

### Limitations

The limitations of this study arise from its reliance on a comprehensive narrative review approach, which synthesises findings from existing literature but may introduce variability due to differences in study methodologies, populations, and outcomes. While HA demonstrates promising therapeutic potential, this review does not provide an exhaustive analysis of its long-term bioactivity or cost-effectiveness in clinical settings. Moreover, the diversity of HA formulations and the lack of standardised protocols for their application in dental implantology pose challenges for deriving uniform conclusions. Also, fewer studies investigate HA’s performance across diverse patient populations, for example, patients with systemic conditions (e.g. osteoporosis and diabetes). The study also does not delve into the comparative performance of various HA formulations (for example, crosslinked vs. non-crosslinked, HA-based composites), which is critical for determining the most effective choices for clinical application. These limitations underscore the need for well-structured clinical trials in the future to substantiate the efficacy and safety of HA across diverse patient cohorts.

## Conclusions and future perspectives

HA plays a crucial role in regenerative tissue engineering owing to its biocompatibility, biodegradability, and bioactivity. It regulates cellular functions such as migration, proliferation, and wound healing through interactions with CD44 and RHAMM receptors. HA’s multifunctionality, including its ability to manage inflammation, promote angiogenesis, and integrate with scaffolds, makes it valuable for bone regeneration, soft tissue healing, and implantology. Despite its rapid biodegradation and limited mechanical strength, HA’s performance as a scaffold can be enhanced through chemical modifications, such as crosslinking and the integration of bioceramics. Advances in HA-based scaffolds have demonstrated significant promise for maxillofacial and bone-related applications, particularly in ARP, augmentation, and peri-implant disease (PID) management. HA supports osteogenesis, improves osseointegration, and aids in the management of PIDs (PiM and peri-implantitis). In addition, HA coatings on implants improve biocompatibility, hydrophilicity, and bacterial resistance, enhancing both peri-implant bone and soft tissue healing. However, challenges remain, including HA’s rapid degradation and potential complications such as skin necrosis and foreign-body reactions. Combining HA with other materials and techniques helps extend its bioactivity and improve clinical outcomes.

Future research on HA in dental implantology should prioritise optimising HA-based scaffolds by enhancing their mechanical properties and degradation rates through crosslinking and material hybridisation. This includes developing HA scaffolds for controlled delivery of drugs or growth factors, as well as exploring their potential in stem cell-based regenerative therapies to expedite healing and tissue regeneration. In addition, longer-term studies are required to further investigate the stability and bioactivity of HA composites, using novel crosslinking techniques and integrating HA with advanced technologies such as 3D printing. Clinical trials should compare HA-based materials with traditional biomaterials to investigate HA’s molecular mechanisms that enhance osseointegration and increase bone density. In managing PIDs, sustained-release HA systems could offer prolonged antibacterial and anti-inflammatory benefits, and further research should examine HA’s synergistic effects with other biomaterials and growth factors. Finally, studies on HA’s molecular interactions with cells and proteins will be crucial to advancing its applications in both soft and hard tissue regeneration, and long-term clinical trials will be required to ensure safety and efficacy.

## Data Availability

Data sharing is not applicable to this article, as no new data were created or analysed in this study.

## Key messages:

1. HA, being a component of the extracellular matrix, can be utilised at various stages of dental implant therapy, including alveolar bone regeneration, implant surface modification, peri-implant disease management, and peri-implant soft tissue healing, with promising results.
2. Advances in scaffolds based on HA have demonstrated significant promise for maxillofacial and bone-related applications, especially in alveolar bone regeneration, preservation, augmentation, and peri-implant disease management.
3. Future research should focus on improving HA’s mechanical properties, enhancing long-term bioactivity, and exploring synergistic combinations of biomaterials; clinical trials are also essential to fully understand HA’s potential in the realm of implant dentistry.

## References

[1] Alqutaibi AY, Algabri R, Ibrahim WI, Alhajj MN, Elawady D. Dental implant planning using artificial intelligence: a systematic review and meta-analysis. J Prosthet Dent. 2024;134: 1619-1629 10.1016/j.prosdent.2024.03.032 38653687

[2] Marx RE. Clinical application of bone biology to mandibular and maxillary reconstruction. Clin Plastic Surg. 1994;21(3):377–92. 10.1016/S0094-1298(20)31014-2 7924135

[3] Alqutaibi AY, Alghauli MA, Aboalrejal A, Mulla AK, Almohammadi AA, Aljayyar AW, et al. Quantitative and qualitative 3D analysis of mandibular lingual concavities: implications for dental implant planning in the posterior mandible. Clin Exp Dent Res. 2024;10(1):e858. 10.1002/cre2.858 38345362 PMC10860544

[4] Alqutaibi AY. CAD-CAM surgically-guided oral implant site expansion and implant placement in severely atrophic maxilla. J Taibah Univ Med Sci. 2020;15(2):153–9. 10.1016/j.jtumed.2020.02.006 32368213 PMC7184207

[5] Rotundo R, Pagliaro U, Bendinelli E, Esposito M, Buti J. Long‐term outcomes of soft tissue augmentation around dental implants on soft and hard tissue stability: a systematic review. Clin Oral Implants Res. 2015;26:123–38. 10.1111/clr.12629 26385627

[6] Begam H, Nandi SK, Kundu B, Chanda A. Strategies for delivering bone morphogenetic protein for bone healing. Mater Sci Eng C Mater Biol Appl. 2017;70:856–69. 10.1016/j.msec.2016.09.074 27770964

[7] Abdalla RIB, Alqutaibi AY, Kaddah A. Does the adjunctive use of platelet-rich plasma to bone graft during sinus augmentation reduce implant failure and complication? Systematic review and meta-analysis. Quintessence Int. 2018;49(2):139-146. 29292406 10.3290/j.qi.a39616

[8] Maekawa S, Cho YD, Kauffmann F, Yao Y, Sugai JV, Zhong X, et al. BMP gene‐immobilization to dental implants enhances bone regeneration. Adv Mater Interfaces. 2022;9(22):2200531. 36387968 10.1002/admi.202200531 PMC9645788

[9] Choi JU, Lee SW, Pangeni R, Byun Y, Yoon I-S, Park JW. Preparation and in vivo evaluation of cationic elastic liposomes comprising highly skin-permeable growth factors combined with hyaluronic acid for enhanced diabetic wound-healing therapy. Acta Biomater. 2017;57:197–215. 10.1016/j.actbio.2017.04.034 28476587

[10] Gravante G, Sorge R, Merone A, Tamisani AM, Di Lonardo A, Scalise A, et al. Hyalomatrix PA in burn care practice: results from a national retrospective survey, 2005 to 2006. Ann Plast Surg. 2010;64(1):69–79. 10.1097/SAP.0b013e31819b3d59 20010411

[11] Kloss FR, Kau T, Heimes D, Kämmerer PW, Kloss-Brandstätter A. Enhanced alveolar ridge preservation with hyaluronic acid-enriched allografts: a comparative study of granular allografts with and without hyaluronic acid addition. Int J Implant Dent. 2024;10(1):42. 10.1186/s40729-024-00559-6 39382763 PMC11465134

[12] Yazan M, Kocyigit I, Atil F, Tekin U, Gonen Z, Onder M. Effect of hyaluronic acid on the osseointegration of dental implants. Br J Oral Maxillofac Surg. 2019;57(1):53–7. 30558816 10.1016/j.bjoms.2018.08.014

[13] Moseley R, Waddington RJ, Embery G. Hyaluronan and its potential role in periodontal healing. Dent Update. 2002;29(3):144–8. 11989392 10.12968/denu.2002.29.3.144

[14] Hosny Elhadidi M, Mohammed Said Ahmed W, Abdelsalam Yossef E. Effect of topical application of hyaluronic acid on stability of immediate loading dental implant in posterior maxilla. Mansoura J Dent. 2020;7(3):11–2. 10.21608/mjd.2020.198722

[15] Salih ARC, Farooqi HMU, Amin H, Karn PR, Meghani N, Nagendran S. Hyaluronic acid: comprehensive review of a multifunctional biopolymer. Futur J Pharm Sci. 2024;10(1):63. 10.1186/s43094-024-00636-y

[16] Boeriu CG, Springer J, Kooy FK, van den Broek LA, Eggink G. Production methods for hyaluronan. Int J Carbohydr Chem. 2013;2013(1):624967. 10.1155/2013/624967

[17] Boas NF. Isolation of hyaluronic acid from the cock’s comb. J Biol Chem. 1949;181(2):573–5. 15407053

[18] Blumberg B, Ogston A, Lowther D, Rogers H. Physicochemical properties of hyaluronic acid formed by Streptococcus haemolyticus. Biochem J. 1958;70(1):1. 10.1042/bj0700001 13584292 PMC1196615

[19] Fletcher E, JH J, RL M. Viscosity studies on hyaluronic acid of synovial fluid in rheumatoid arthritis and osteoarthritis. Clin Sci. 1955;14(4):653–60. 13270449

[20] Kendall FE, Heidelberger M, Dawson MH. A serologieally inactive polysaccharide elaborated by mucoid strains of group A hemolytie streptococcus. 1937;118(1):61-69. 10.1016/S0021-9258(18)74517-1

[21] Weissmann B, Meyer K. The structure of hyalobiuronic acid and of hyaluronic acid from umbilical Cord1, 2. J Am Chem Soc. 1954;76(7):1753–7. 10.1021/ja01636a010

[22] Necas J, Bartosikova L, Brauner P, Kolar J. Hyaluronic acid (hyaluronan): a review. Vet Med. 2008;53(8):397–411. 10.17221/1930-VETMED

[23] Balazs EA. Ultrapure hyaluronic acid and the use thereof. Google Patents; 1979. United States https://patents.google.com/patent/US4141973A

[24] Miller D, Stegmann R. Use of Na-hyaluronate in anterior segment eye surgery. J Cataract Refract Surg. 1980;6(1):13–5. 10.1016/S0146-2776(80)80097-8 7358568

[25] Regnault F, Bregeat P. Treatment of severe cases of retinal detachment with highly viscous hyaluronic acid. Mod Probl Ophthalmol. 1974;12:378–83. 4424797

[26] Jones AC, Pattrick M, Doherty S, Doherty M. Intra-articular hyaluronic acid compared to intra-articular triamcinolone hexacetonide in inflammatory knee osteoarthritis. Osteoarthritis Cartilage. 1995;3(4):269–73. 10.1016/S1063-4584(05)80018-4 8689462

[27] Pavicic T, Gauglitz GG, Lersch P, Schwach-Abdellaoui K, Malle B, Korting HC, et al. Efficacy of cream-based novel formulations of hyaluronic acid of different molecular weights in anti-wrinkle treatment. J Drugs Dermatol. 2011;10(9):990–1000. 22052267

[28] Hellström S, Laurent C. Hyaluronan and healing of tympanic membrane perforations. An experimental study. Acta Oto-Laryngol Suppl. 1987;104(sup442):54–61. 10.3109/00016488709102840 3481167

[29] Duranti F, Salti G, Bovani B, Calandra M, Rosati ML. Injectable hyaluronic acid gel for soft tissue augmentation: a clinical and histological study. Dermatol Surg. 1998;24(12):1317–25. 10.1111/j.1524-4725.1998.tb00007.x 9865196

[30] Camber O, Edman P. Sodium hyaluronate as an ophthalmic vehicle: some factors governing its effect on the ocular absorption of pilocarpine. Curr Eye Res. 1989;8(6):563–7. 2743795 10.3109/02713688908995755

[31] Benedetti L, Topp E, Stella V. Microspheres of hyaluronic acid esters – fabrication methods and in vitro hydrocortisone release. J Control Release. 1990;13(1):33–41. 10.1016/0168-3659(90)90072-2

[32] Pagnacco A, Vangelisti R, Erra C, Poma A. Double-blind clinical trial vs. placebo of a new sodium-hyaluronate-based gingival gel. Attualita Terapeutica Internazionale. 1997;15:1–7.

[33] Miglani A, Vishnani R, Reche A, Buldeo J, Wadher B. Hyaluronic acid: exploring its versatile applications in dentistry. Cureus. 2023;15(10): e46349. 37920632 10.7759/cureus.46349 PMC10618852

[34] Sapna N. Clinical evaluation of hyaluronan gel (Gengigel) as a toipical application in the treatment of gingivitis. Rajiv Gandhi University of Health Sciences. Journal of Investigative and Clinical Dentistry Wiley-Blackwell: Hoboken, New Jersey, USA; 2006.

[35] Petersen S, Kaule S, Teske M, Minrath I, Schmitz K-P, Sternberg K. Development and in vitro characterization of hyaluronic acid‐based coatings for implant‐associated local drug delivery systems. J Chem. 2013;2013(1):587875. 10.1155/2013/587875

[36] Chircov C, Grumezescu AM, Bejenaru LE. Hyaluronic acid-based scaffolds for tissue engineering. Rom J Morphol Embryol. 2018;59(1):71–6. 29940614

[37] Yun J, Lee J, Ha CW, Park SJ, Kim S, Koo KT, et al. The effect of 3‐D printed polylactic acid scaffold with and without hyaluronic acid on bone regeneration. J Periodontol. 2022;93(7):1072–82. 10.1002/JPER.21-0428 34773704

[38] Lee JH, Kim J, Baek H-R, Lee KM, Seo J-H, Lee H-K, et al. Fabrication of an rhBMP-2 loaded porous β-TCP microsphere-hyaluronic acid-based powder gel composite and evaluation of implant osseointegration. J Mater Sci Mater Med. 2014;25:2141–51. 24928668 10.1007/s10856-014-5250-0 PMC4137170

[39] Ibraheem W, Jedaiba W, Alnami A, Hussain Baiti L, Ali Manqari S, Bhati A, et al. Efficacy of hyaluronic acid gel and spray in healing of extraction wound: a randomized controlled study. Eur Rev Med Pharmacol Sci. 2022;26(10):3444–9. 35647824 10.26355/eurrev_202205_28838

[40] Koray M, Ofluoglu D, Onal E, Ozgul M, Ersev H, Yaltirik M, et al. Efficacy of hyaluronic acid spray on swelling, pain, and trismus after surgical extraction of impacted mandibular third molars. Int J Oral Maxillofac Surg. 2014;43(11):1399–403. 10.1016/j.ijom.2014.05.003 24924267

[41] Sánchez-Fernández E, Magán-Fernández A, O’Valle F, Bravo M, Mesa F. Hyaluronic acid reduces inflammation and crevicular fluid IL-1β concentrations in peri-implantitis: a randomized controlled clinical trial. J Periodontal Implant Sci. 2020;51(1):63. 10.5051/jpis.1903660183 PMC7920839 33634616

[42] Genovesi A, Barone A, Toti P, Covani U. The efficacy of 0.12% chlorhexidine versus 0.12% chlorhexidine plus hyaluronic acid mouthwash on healing of submerged single implant insertion areas: a short‐term randomized controlled clinical trial. Int J Dent Hyg. 2017;15(1):65–72. 26084554 10.1111/idh.12158

[43] Avila-Ortiz G, Elangovan S, Kramer K, Blanchette D, Dawson D. Effect of alveolar ridge preservation after tooth extraction: a systematic review and meta-analysis. J Dent Res. 2014;93(10):950–8. 10.1177/0022034514541127 24966231 PMC4293706

[44] Iasella JM, Greenwell H, Miller RL, Hill M, Drisko C, Bohra AA, et al. Ridge preservation with freeze‐dried bone allograft and a collagen membrane compared to extraction alone for implant site development: a clinical and histologic study in humans. J Periodontol. 2003;74(7):990–9. 12931761 10.1902/jop.2003.74.7.990

[45] Kloss FR, Offermanns V, Kloss‐Brandstätter A. Comparison of allogeneic and autogenous bone grafts for augmentation of alveolar ridge defects – a 12‐month retrospective radiographic evaluation. Clin Oral Implants Res. 2018;29(11):1163–75. 10.1111/clr.13380 30303581 PMC6282851

[46] Kämmerer PW, Tunkel J, Götz W, Würdinger R, Kloss F, Pabst A. The allogeneic shell technique for alveolar ridge augmentation: a multicenter case series and experiences of more than 300 cases. Int J Implant Dent. 2022;8(1):48. 36316597 10.1186/s40729-022-00446-y PMC9622968

[47] Majzoub J, Ravida A, Starch-Jensen T, Tattan M, Del Amo FS-L. The influence of different grafting materials on alveolar ridge preservation: a systematic review. J Oral Maxillofac Res. 2019;10(3): e6. 10.5037/jomr.2019.10306 31620268 PMC6788425

[48] Majidinia M, Sadeghpour A, Yousefi B. The roles of signaling pathways in bone repair and regeneration. J Cell Physiol. 2018;233(4):2937–48. 28590066 10.1002/jcp.26042

[49] Zhai P, Peng X, Li B, Liu Y, Sun H, Li X. The application of hyaluronic acid in bone regeneration. Int J Biol Macromol. 2020;151:1224–39. 10.1016/j.ijbiomac.2019.10.169 31751713

[50] Rao NV. Hyaluronic acid–based hydrogels for tissue engineering. In: Biomaterials for organ and tissue regeneration. Hindawi Publishing Corporation. Elsevier: Cairo, Egypt. 2020;551–65.

[51] Husseini B, Friedmann A, Wak R, Ghosn N, Khoury G, Ghoul TE, et al. Clinical and radiographic assessment of cross-linked hyaluronic acid addition in demineralized bovine bone based alveolar ridge preservation: a human randomized split-mouth pilot study. J Stomatol Oral Maxillofac Surg. 2023;124(4):101426. 10.1016/j.jormas.2023.101426 36801259

[52] Aldeen Abdelzaher KM. Clinical and radiographic assessment of xenogenic bone graft with or without hyaluronic acid for post extraction socket preservation (randomized clinical trial). Future. 2022;8(2):101–8.

[53] Abdullah AAB, Ali HE-DM, Al-Ashmawy MMM, Mwafey IM. Volumetric and histological evaluation of Osteon II collagen with hyaluronic acid versus sticky bone graft in three dimensional socket preservation. Egypt Dent J. 2020;66(3-ِJuly (Oral Surgery)):1483–94. 10.21608/edj.2020.26333.1081

[54] Ronsivalle V, Santonocito S, Giudice R, Bocchieri S, Didomenico S, Cicciù M. The role of hyaluronic acid in alveolar ridge preservation: a systematic review of its biological and regenerative potential according to PRISMA guidelines and the Cochrane handbook. Biomedicines. 2025;13(2):451. 10.3390/biomedicines13020451 40002864 PMC11853319

[55] Helal MH, Sheta MS, Alsherif AA, Hassan MA, Aboushelib MN, Ghouraba RF. The effectiveness of hyaluronic acid on prefabricated CAD CAM bone blocks for ridge augmentation: a split mouth study. Clin Implant Dent Relat Res. 2025;27(2):e70035. 10.1111/cid.70035 40231349

[56] Abaza G, Abdel Gaber HK, Afifi NS, Adel‐Khattab D. Injectable platelet rich fibrin versus hyaluronic acid with bovine derived xenograft for alveolar ridge preservation. A randomized controlled clinical trial with histomorphometric analysis. Clin Implant Dent Relat Res. 2024;26(1):88–102. 37905704 10.1111/cid.13289

[57] Lee JB, Chu S, Ben Amara H, Song HY, Son MJ, Lee J, et al. Effects of hyaluronic acid and deproteinized bovine bone mineral with 10% collagen for ridge preservation in compromised extraction sockets. J Periodontol. 2021;92(11):1564–75. 10.1002/JPER.20-0832 33484160

[58] Kang H-J, Park S-S, Saleh T, Ahn K-M, Lee B-T. In vitro and in vivo evaluation of Ca/P-hyaluronic acid/gelatin based novel dental plugs for one-step socket preservation. Mater Design. 2020;194:108891.

[59] Kloss FR, Kämmerer PW, Kloss-Brandstätter A. First clinical case report of a xenograft–allograft combination for alveolar ridge augmentation using a bovine bone substitute material with hyaluronate (Cerabone^®^ Plus) combined with allogeneic bone granules (Maxgraft^®^). J Clin Med. 2023;12(19):6214. 10.3390/jcm12196214 37834860 PMC10573600

[60] El Halawani GN, Khalil MM. Evaluation of melatonin and hyaluronic acid in maxillary sinus augmentation (A randomized controlled clinical trial). Alex Dent J. 2022;47(3):80–7.

[61] Zhao N, Qin L, Liu Y, Zhai M, Li D. Improved new bone formation capacity of hyaluronic acid-bone substitute compound in rat calvarial critical size defect. BMC Oral Health. 2024;24(1):994. 10.1186/s12903-024-04679-8 39182066 PMC11344309

[62] Darby I. Risk factors for periodontitis & peri‐implantitis. Periodontology 2000. 2022;90(1):9–12. 35913624 10.1111/prd.12447 PMC9804916

[63] Pontoriero R, Tonelli M, Carnevale G, Mombelli A, Nyman SR, Lang NP. Experimentally induced peri‐implant mucositis. A clinical study in humans. Clin Oral Implants Res. 1994;5(4):254–9. 10.1034/j.1600-0501.1994.050409.x 7640340

[64] Schimmel M, Mombelli A. Experimental mucositis and experi-mental gingivitis in persons aged 70 or over. Clinical and biological responses. Clinical Oral Implants Research. 2016;28(8):1005–1012. 10.1111/clr.12912 27333829 PMC5599942

[65] Berglundh T, Armitage G, Araujo MG, Avila‐Ortiz G, Blanco J, Camargo PM, et al. Peri‐implant diseases and conditions: consensus report of workgroup 4 of the 2017 world workshop on the classification of periodontal and peri‐implant diseases and conditions. J Periodontol. 2018;89:S313–8. 10.1002/JPER.17-0739 29926955

[66] Schwarz F, Derks J, Monje A, Wang HL. Peri‐implantitis. J Clin Periodontol. 2018;45:S246–66. 29926484 10.1111/jcpe.12954

[67] Herrera D, Berglundh T, Schwarz F, Chapple I, Jepsen S, Sculean A, et al. Prevention and treatment of peri‐implant diseases – the EFP S3 level clinical practice guideline. J Clin Periodontol. 2023;50:4–76. 10.1111/jcpe.13823 37271498

[68] Monje A, Salvi GE. Diagnostic methods/parameters to monitor peri‐implant conditions. Periodontology 2000. 2024;95: 20-39 38923148 10.1111/prd.12584

[69] Sahrmann P, Kühl S, Dagassan‐Berndt D, Bornstein MM, Zitzmann NU. Radiographic assessment of the peri‐implant site. Periodontology 2000. 2024;95: 70-86. 10.1111/prd.12577 38951952

[70] Salvi GE, Ramseier CA. Efficacy of patient‐administered mechanical and/or chemical plaque control protocols in the management of peri‐implant mucositis. A systematic review. J Clin Periodontol. 2015;42:S187–201. 25495416 10.1111/jcpe.12321

[71] Scully MF, Kakkar VV, Goodwin CA, O’Regan M. Inhibition of fibrinolytic activity by hyaluronan and its alcohol ester derivatives. Thromb Res. 1995;78(3):255–8. 10.1016/0049-3848(95)90876-H 7631306

[72] Shirakata Y, Nakamura T, Kawakami Y, Imafuji T, Shinohara Y, Noguchi K, et al. Healing of buccal gingival recessions following treatment with coronally advanced flap alone or combined with a cross‐linked hyaluronic acid gel. An experimental study in dogs. J Clin Periodontol. 2021;48(4):570–80. 10.1111/jcpe.13433 33513277 PMC8248173

[73] Shirakata Y, Imafuji T, Nakamura T, Shinohara Y, Iwata M, Setoguchi F, et al. Cross‐linked hyaluronic acid gel with or without a collagen matrix in the treatment of class III furcation defects: a histologic and histomorphometric study in dogs. J Clin Periodontol. 2022;49(10):1079–89. 35817414 10.1111/jcpe.13694 PMC9796036

[74] Iorio-Siciliano V, Marasca D, Mauriello L, Vaia E, Stratul S-I, Ramaglia L. Treatment of peri-implant mucositis using spermidine and calcium chloride as local adjunctive delivery to non-surgical mechanical debridement: a double-blind randomized controlled clinical trial. Clin Oral Investig. 2024;28(10):1–11. 10.1007/s00784-024-05924-8 39304548

[75] Lopez M, Manzulli N, D’Angelo A, Candotto V, Casale M, Lauritano D. The use of hyaluronic acid as an adjuvant in the management of mucositis. J Biol Regul Homeost Agents. 2017;31(4 Suppl 2):115–8. 29202570

[76] Parfenova LV, Galimshina ZR, Gil’fanova GU, Alibaeva EI, Danilko KV, Pashkova TM, et al. Hyaluronic acid bisphosphonates as antifouling antimicrobial coatings for PEO-modified titanium implants. Surf Interfaces. 2022;28:101678. 10.1016/j.surfin.2021.101678

[77] Derks J, Tomasi C. Peri‐implant health and disease. A systematic review of current epidemiology. J Clin Periodontol. 2015;42:S158–71. 10.1111/jcpe.12334 25495683

[78] Bohara S, Suthakorn J. Surface coating of orthopedic implant to enhance the osseointegration and reduction of bacterial colonization: a review. Biomater Res. 2022;26(1):26. 35725501 10.1186/s40824-022-00269-3 PMC9208209

[79] Sanz M, Chapple IL, Periodontology* WGotVEWo. Clinical research on peri‐implant diseases: consensus report of W orking G roup 4. J Clin Periodontol. 2012;39:202–6. 10.1111/j.1600-051X.2011.01837.x 22533957

[80] Sutherland IW. Novel and established applications of microbial polysaccharides. Trends Biotechnol. 1998;16(1):41–6. 10.1016/S0167-7799(97)01139-6 9470230

[81] Moreland LW. Intra-articular hyaluronan (hyaluronic acid) and hylans for the treatment of osteoarthritis: mechanisms of action. Arthritis Res Ther. 2003;5:1–14. 10.1186/ar623 PMC165033 12718745

[82] Chen WJ. Functions of hyaluronan in wound repair. Hyaluronan. 2002:147–56. 10.1046/j.1524-475x.1999.00079.x 10231509

[83] Manzanares D, Monzon M-E, Savani RC, Salathe M. Apical oxidative hyaluronan degradation stimulates airway ciliary beating via RHAMM and RON. Am J Respir Cell Mol Biol. 2007;37(2):160–8. 10.1165/rcmb.2006-0413OC 17395888 PMC1976543

[84] Soriano‐Lerma A, Magán‐Fernández A, Gijón J, Sánchez‐Fernández E, Soriano M, García‐Salcedo JA, et al. Short‐term effects of hyaluronic acid on the subgingival microbiome in peri‐implantitis: a randomized controlled clinical trial. J Periodontol. 2020;91(6):734–45. 31577041 10.1002/JPER.19-0184

[85] Zhou Z, Zhang Q, Wang Y. Preparation and characterization of antibacterial and anti-inflammatory hyaluronic acid-chitosan-dexamethasone hydrogels for peri-implantitis repair. J Biomater Appl. 2022;36(7):1141–50. 10.1177/08853282211047939 34605300

[86] Rakašević D, Šćepanović M, Mijailović I, Mišić T, Janjić B, Soldatović I, et al. Reconstructive peri-implantitis therapy by using bovine bone substitute with or without hyaluronic acid: a randomized clinical controlled pilot study. J Funct Biomater. 2023;14(3):149. 36976073 10.3390/jfb14030149 PMC10053283

[87] López-Valverde N, López-Valverde A, Blanco Rueda JA. Role of hyaluronic acid in the treatment of peri-implant diseases: results of a meta-analysis. Front Oral Health. 2025;6:1564599. 10.3389/froh.2025.1564599 40376205 PMC12078234

[88] Friedmann A, Jung R, Bilhan H, Ghawi-Begovic HA, Kauffmann F, Diehl D. Reconstructive surgical therapy of peri-implant defects with ribose cross-linked collagen matrix and crosslinked hyaluronic acid–a prospective case series. Clin Oral Investig. 2024;28(10):536. 10.1007/s00784-024-05942-6 PMC11415415 39302523

[89] Alharbi MS, Alshehri FA. High molecular weight hyaluronic acid reduces the expression of virulence genes fima, mfa1, haga, rgpa, and kgp in the oral pathogen Porphyromonas gingivalis. Pharmaceutics. 2022;14(8):1628. 10.3390/pharmaceutics14081628 36015254 PMC9415305

[90] Lopez M, Manzulli N, D’Angelo A, Lauritano D, Papalia R, Candotto V. The use of hyaluronic acid as an adjuvant in the management of peri-implantitis. J Biol Regul Homeost Agents. 2017;31(4 Suppl 2):123–7. 29202572

[91] Yumashev A, Karapetyan A, Garnova N, Berestova A. Characteristics of biocompatible coatings on dental implants. J Global Pharma Technol. 2020;12(1):30.

[92] Schulz MC, Korn P, Stadlinger B, Range U, Möller S, Becher J, et al. Coating with artificial matrices from collagen and sulfated hyaluronan influences the osseointegration of dental implants. J Mater Sci Mater Med. 2014;25:247–58. 10.1007/s10856-013-5066-3 24113890

[93] Dreifke MB, Ebraheim NA, Jayasuriya AC. Investigation of potential injectable polymeric biomaterials for bone regeneration. J Biomed Mater Res Part A. 2013;101(8):2436–47. 10.1002/jbm.a.34521 PMC4135428 23401336

[94] Schanté CE, Zuber G, Herlin C, Vandamme TF. Chemical modifications of hyaluronic acid for the synthesis of derivatives for a broad range of biomedical applications. Carbohydr Polym. 2011;85(3):469–89. 10.1016/j.carbpol.2011.03.019

[95] Mendes RM, Silva GA, Lima MF, Calliari MV, Almeida AP, Alves JB, et al. Sodium hyaluronate accelerates the healing process in tooth sockets of rats. Archiv Oral Biol. 2008;53(12):1155–62. 10.1016/j.archoralbio.2008.07.001 18692778

[96] Hamdy M, Nasr S, Mater N, ElSayed O. Assessment of osseointegration properties of hyaluronic acid coated titanium implant versus sandblasted large thread acid etched implants in posterior maxilla. A randomized clinical trial. MSA Dent J. 2024;3(4):1–7.

[97] Abdulghafor MA, Amin ZM. The impact of hyaluronic acid coating on polyether ether ketone dental implant surface: an in vitro analysis. Saudi Dent J. 2024;36(10):1326–32. 10.1016/j.sdentj.2024.07.012 39525932 PMC11544272

[98] Ganesh S, Gheena S, Madhu K. Commercially pure titanium implants with selenium and hyaluronic acid coating for dental applications. Cureus. 2024;16(1):e52984. 38406106 10.7759/cureus.52984 PMC10893994

[99] Del Olmo JA, Alonso JM, Martínez VS, Ruiz-Rubio L, González RP, Vilas-Vilela JL, et al. Biocompatible hyaluronic acid-divinyl sulfone injectable hydrogels for sustained drug release with enhanced antibacterial properties against Staphylococcus aureus. Mater Sci Eng C. 2021;125:112102. 10.1016/j.msec.2021.112102 33965111

[100] Jin H, Wang J, Tian L, Gao M, Zhao J, Ren L. Recent advances in emerging integrated antifouling and anticorrosion coatings. Mater Des. 2022;213:110307. 10.1016/j.matdes.2021.110307

[101] Zamboni F, Okoroafor C, Ryan MP, Pembroke JT, Strozyk M, Culebras M, et al. On the bacteriostatic activity of hyaluronic acid composite films. Carbohydr Polym. 2021;260:117803. 10.1016/j.carbpol.2021.117803 33712151

[102] Sánchez-Bodón J, Andrade del Olmo J, Alonso JM, Moreno-Benítez I, Vilas-Vilela JL, Pérez-Álvarez L. Bioactive coatings on titanium: a review on hydroxylation, self-assembled monolayers (SAMs) and surface modification strategies. Polymers. 2021;14(1):165. 35012187 10.3390/polym14010165 PMC8747097

[103] Del Olmo JA, Alonso JM, Sáez-Martínez V, Benito-Cid S, Pérez-González R, Vilas-Vilela JL, et al. Hyaluronic acid-based hydrogel coatings on Ti6Al4V implantable biomaterial with multifunctional antibacterial activity. Carbohydr Polym. 2023;301:120366. 36446504 10.1016/j.carbpol.2022.120366

[104] Hu X, Neoh K-G, Shi Z, Kang E-T, Poh C, Wang W. An in vitro assessment of titanium functionalized with polysaccharides conjugated with vascular endothelial growth factor for enhanced osseointegration and inhibition of bacterial adhesion. Biomaterials. 2010;31(34):8854–63. 10.1016/j.biomaterials.2010.08.006 20800276

[105] Valverde A, Pérez-Álvarez L, Ruiz-Rubio L, Olivenza MAP, Blanco MBG, Díaz-Fuentes M, et al. Antibacterial hyaluronic acid/chitosan multilayers onto smooth and micropatterned titanium surfaces. Carbohydr Polym. 2019;207:824–33. 10.1016/j.carbpol.2018.12.039 30600071

[106] Zhong X, Song Y, Yang P, Wang Y, Jiang S, Zhang X, et al. Titanium surface priming with phase-transited lysozyme to establish a silver nanoparticle-loaded chitosan/hyaluronic acid antibacterial multilayer via layer-by-layer self-assembly. PLoS One. 2016;11(1):e0146957. 26783746 10.1371/journal.pone.0146957 PMC4718720

[107] Yurttutan E, Dereci Ö, Karagöz MA. Biomechanical and histologic evaluation of osseointegration of titanium dental implants modified by various combinations of sandblasting, acid-etching, hydroxyapatite, and hyaluronic acid coating techniques. Int J Oral Maxillofac Implants. 2023;38(3): 583-590. 10.11607/jomi.9935 37279232

[108] Shamma MM, Ayad SS, El-dibany RM, Nagui DA. Evaluation of the effect of hyaluronic acid mixed with biphasic calcium phosphate on bone healing around dental implants (experimental study). Alexandria Dent J. 2017;42(1):104–7. 10.21608/adjalexu.2017.57868

[109] Ibrahim WS, Mohammed FI, Reda HM. Evaluation of the effect of hyaluronic acid gel loaded with simvastatin on the osseointegration and stability of the dental implants. Al-Azhar Dent J Girls. 2023;10(1):393–400.

[110] Bassiouny G, Abu Brika M, Ezzat M, Hommos A. Evaluation of the efficacy of flowable leukocyte and platelet–rich fibrin versus hyaluronic acid as bioactive implant coatings (randomized controlled clinical trial). Egypt Dent J. 2024;70(2):1339–53. 10.21608/edj.2024.267529.2920

[111] Chai WL, Razali M, Ngeow WC. Dimension and structures of biological seal of peri-implant tissues. Dent Implantol Biomater. 2016;39: 39-62. 10.5772/63950

[112] Favia G, Mariggio M, Maiorano F, Cassano A, Capodiferro S, Ribatti D. Accelerated wound healing of oral soft tissues and angiogenic effect induced by a pool of aminoacids combined to sodium hyaluronate (AMINOGAM). J Biol Regul Homeost Agents. 2008;22(2):109–16. 18597703

[113] Cohen IK, Die-Gelmann RF, Lindblad WJ, Hugo NE. Wound healing: biochemical and clinical aspects. Plast Reconstr Surg. 1992;90(5):926. 10.1097/00006534-199211000-00034

[114] Brown LF, Yeo K, Berse B, Yeo T-K, Senger DR, Dvorak HF, et al. Expression of vascular permeability factor (vascular endothelial growth factor) by epidermal keratinocytes during wound healing. J Exp Med. 1992;176(5):1375–9. 1402682 10.1084/jem.176.5.1375 PMC2119412

[115] Kim J-W, Han Y-S, Lee H-M, Kim J-K, Kim Y-J. Effect of morphological characteristics and biomineralization of 3D-printed gelatin/hyaluronic acid/hydroxyapatite composite scaffolds on bone tissue regeneration. Int J Mol Sci. 2021;22(13):6794. 10.3390/ijms22136794 34202759 PMC8267715

[116] Partsch G, Schwarzer C, Neumüller J, Dunky A, Petera P, Bröll H, et al. Modulation of the migration and chemotaxis of PMN cells by hyaluronic acid. Zeitschrift fur Rheumatologie. 1989;48(3):123–8. 2551105

[117] Ouasti S, Donno R, Cellesi F, Sherratt MJ, Terenghi G, Tirelli N. Network connectivity, mechanical properties and cell adhesion for hyaluronic acid/PEG hydrogels. Biomaterials. 2011;32(27):6456–70. 21680016 10.1016/j.biomaterials.2011.05.044

[118] Fallacara A, Baldini E, Manfredini S, Vertuani S. Hyaluronic acid in the third millennium. Polymers. 2018;10(7):701. 10.3390/polym10070701 30960626 PMC6403654

[119] Scholz N. Personalised medicine: the right treatment for the right person at the right time. European Parliamentary Research Service (EPRS), Members’ Research Service, European Parliament: 2015.

[120] Mariggiò MA, Cassano A, Vinella A, Vincenti A, Fumarulo R, Muzio LL, et al. Enhancement of fibroblast proliferation, collagen biosynthesis and production of growth factors as a result of combining sodium hyaluronate and aminoacids. Int J Immunopathol Pharmacol. 2009;22(2):485–92. 10.1177/039463200902200225 19505400

[121] Tammi MI, Day AJ, Turley EA. Hyaluronan and homeostasis: a balancing act. J Biol Chem. 2002;277(7):4581–4. 10.1074/jbc.R100037200 11717316

[122] Mesa F, Aneiros J, Cabrera A, Bravo M, Caballero T, Revelles F, et al. Antiproliferative effect of topic hyaluronic acid gel. Study in gingival biopsies of patients with periodontal disease. Histol Histopathol; 2002;17: 747–753. 12168783 10.14670/HH-17.747

[123] Litwiniuk M, Krejner A, Speyrer MS, Gauto AR, Grzela T. Hyaluronic acid in inflammation and tissue regeneration. Wounds. 2016; 28(3):78–88. 26978861

[124] S.p.A. PD. Aminogam. 2021. Available from: https://aminogam.com/ [cited 28-October-2024]

[125] Canciani E, Sirello R, Pellegrini G, Henin D, Perrotta M, Toma M, et al. Effects of vitamin and amino acid-enriched hyaluronic acid gel on the healing of oral mucosa: in vivo and in vitro study. Medicina. 2021;57(3):285. 10.3390/medicina57030285 33803814 PMC8003116

[126] Capodiferro S, Tempesta A, Bucci S, Maiorano E, Favia G, Limongelli L. Aminogam^®^ gel allows faster wound healing after oral surgery by formation of mature connective tissue with low vascular density and reducing inflammatory infiltration. A retrospective study on 580 cases with histological and confocal laser investigation. Appl Sci. 2020;10(3):1105.

[127] Bawankar PV, Tuli P, Kolte AP, Kolte RA. Evaluation of the efficacy of microneedling alone and in combination with injectable hyaluronic acid in augmentation of peri-implant soft tissues: a randomized controlled trial. J Indian Soc Periodontol. 2024;28(6):643–50. 10.4103/jisp.jisp_158_24 40313337 PMC12043223

[128] Pilipchuk SP, Plonka AB, Monje A, Taut AD, Lanis A, Kang B, et al. Tissue engineering for bone regeneration and osseointegration in the oral cavity. Dent Mater. 2015;31(4):317–38. 25701146 10.1016/j.dental.2015.01.006 PMC4376374

[129] Kostadinova M, Raykovska M, Simeonov R, Lolov S, Mourdjeva M. Recent advances in bone tissue engineering: enhancing the potential of mesenchymal stem cells for regenerative therapies. Curr Issues Mol Biol. 2025;47(4):287. 10.3390/cimb47040287 40699686 PMC12025986

[130] Zhang C, Chen Z, Liu J, Wu M, Yang J, Zhu Y, et al. 3D-printed pre-tapped-hole scaffolds facilitate one-step surgery of predictable alveolar bone augmentation and simultaneous dental implantation. Compos Part B Eng. 2022;229:109461.

[131] Wong SK, Yee MMF, Chin K-Y, Ima-Nirwana S. A review of the application of natural and synthetic scaffolds in bone regeneration. J Funct Biomater. 2023;14(5):286. 10.3390/jfb14050286 37233395 PMC10218951

[132] Roseti L, Parisi V, Petretta M, Cavallo C, Desando G, Bartolotti I, et al. Scaffolds for bone tissue engineering: state of the art and new perspectives. Mater Sci Eng C Mater Biol Appl. 2017;78:1246–62. 28575964 10.1016/j.msec.2017.05.017

[133] D’Albis G, D’Albis V, Palma M, Plantamura M, Nizar AK. Use of hyaluronic acid for regeneration of maxillofacial bones. Genesis. 2022;60(8–9):e23497. 35950678 10.1002/dvg.23497

[134] Hwang HS, Lee C-S. Recent progress in hyaluronic-acid-based hydrogels for bone tissue engineering. Gels. 2023;9(7):588. 10.3390/gels9070588 37504467 PMC10379028

[135] AlHowaish NA, AlSudani DI, AlMuraikhi NA. Evaluation of a hyaluronic acid hydrogel (Restylane Lyft) as a scaffold for dental pulp regeneration in a regenerative endodontic organotype model. Odontology. 2022;110(4):726–34. 35471745 10.1007/s10266-022-00710-y

[136] Seliktar D. Designing cell-compatible hydrogels for biomedical applications. Science. 2012;336(6085):1124–8. 10.1126/science.1214804 22654050

[137] Alshehadat SA, Thu HA, Hamid SSA, Nurul AA, Rani SA, Ahmad A. Scaffolds for dental pulp tissue regeneration: a review. Int Dent Med J Adv Res. 2016;2(1):1–12.

[138] Hamlet SM, Vaquette C, Shah A, Hutmacher DW, Ivanovski S. 3‐Dimensional functionalized polycaprolactone‐hyaluronic acid hydrogel constructs for bone tissue engineering. J Clin Periodontol. 2017;44(4):428–37. 28032906 10.1111/jcpe.12686

[139] Subramaniam S, Fang Y-H, Sivasubramanian S, Lin F-H, Lin C-p. Hydroxyapatite-calcium sulfate-hyaluronic acid composite encapsulated with collagenase as bone substitute for alveolar bone regeneration. Biomaterials. 2016;74:99–108. 10.1016/j.biomaterials.2015.09.044 26454048

[140] Zanchetta P, Lagarde N, Uguen A, Marcorelles P. Mixture of hyaluronic acid, chondroitin 6 sulphate and dermatan sulphate used to completely regenerate bone in rat critical size defect model. J Cranio-Maxillofac Surg. 2012;40(8):783–7. 10.1016/j.jcms.2012.02.011 22464550

[141] Azhar IS, Nariswari VS, Kusumawardhani DP, Maksum MA. Combination of hDPSCs and oxysterol in Hyaluronic Acid scaffolds for Dental Implant Therapy: a narrative review. J Int Oral Health. 2022;14(5):440–6. 10.4103/jioh.jioh_299_21

[142] Li S, De Wijn JR, Li J, Layrolle P, De Groot K. Macroporous biphasic calcium phosphate scaffold with high permeability/porosity ratio. Tissue Eng. 2003;9(3):535–48. 12857421 10.1089/107632703322066714

[143] Park S, Huang NW, Wong CX, Pan J, Albakr L, Gu J, et al. Microstructured hyaluronic acid hydrogel for tooth germ bioengineering. Gels. 2021;7(3):123. 34449604 10.3390/gels7030123 PMC8395742

[144] Pan H, Han JJ, Park Y-D, Cho TH, Hwang SJ. Effect of sustained release of rhBMP-2 from dried and wet hyaluronic acid hydrogel carriers compared with direct dip coating of rhBMP-2 on peri-implant osteogenesis of dental implants in canine mandibles. J Cranio-Maxillofac Surg. 2016;44(2):116–25. 10.1016/j.jcms.2015.11.018 26732636

[145] Park SH, Park JY, Ji YB, Ju HJ, Min BH, Kim MS. An injectable click-crosslinked hyaluronic acid hydrogel modified with a BMP-2 mimetic peptide as a bone tissue engineering scaffold. Acta Biomater. 2020;117:108–20. 10.1016/j.actbio.2020.09.013 32927087

[146] Miranda DG, Malmonge SM, Campos DM, Attik NG, Grosgogeat B, Gritsch K. A chitosan‐hyaluronic acid hydrogel scaffold for periodontal tissue engineering. J Biomed Mater Res Part B Appl Biomater. 2016;104(8):1691–702. 10.1002/jbm.b.33516 26344054

[147] Chrepa V, Austah O, Diogenes A. Evaluation of a commercially available hyaluronic acid hydrogel (Restylane) as injectable scaffold for dental pulp regeneration: an in vitro evaluation. J Endod. 2017;43(2):257–62. 10.1016/j.joen.2016.10.026 28041686

[148] Jung YS, Ye JR, Kwack KH, Lee M-H, Kweon D-K, Chae YK, et al. Bilayer cellulose-coated hyaluronic acid-based scaffold for accelerating oral wound healing. Cellulose. 2024:31;1–14.

[149] Sabu A, Kaarthikeyan G, Eswaramoorthy R, Priyangha P. Development of a Cissus quadrangularis-doped extracellular matrix and a hyaluronic acid-incorporated scaffold for periodontal regeneration: an in vitro study. Cureus. 2024;16(3): e56507. 38646344 10.7759/cureus.56507 PMC11026305

[150] ElShammaa IM, Ghoraba SF, Shoukheba MY. Effect of hyaluronic acid with a collagen sponge scaffold in the treatment of intra-bony defects of chronic periodontitis. Tanta Dent J. 2023;20(3):224–32. 10.4103/tdj.tdj_10_23

[151] Lee S-w, Kim J, Do M, Namkoong E, Lee H, Ryu JH, et al. Developmental role of hyaluronic acid and its application in salivary gland tissue engineering. Acta Biomater. 2020;115:275–87. 10.1016/j.actbio.2020.08.030 32853803

[152] Sieger D, Korzinskas T, Jung O, Stojanovic S, Wenisch S, Smeets R, et al. The addition of high doses of hyaluronic acid to a biphasic bone substitute decreases the proinflammatory tissue response. Int J Mol Sci. 2019;20(8):1969. 31013636 10.3390/ijms20081969 PMC6515558

[153] Rőszer T. Understanding the mysterious M2 macrophage through activation markers and effector mechanisms. Mediators Inflamm. 2015;2015(1):816460. 26089604 10.1155/2015/816460 PMC4452191

[154] Bertl K, Gotfredsen K, Jensen SS, Bruckmann C, Stavropoulos A. Adverse reaction after hyaluronan injection for minimally invasive papilla volume augmentation. A report on two cases. Clin Oral Implants Res. 2017;28(7):871–6. 10.1111/clr.12892 27252126

[155] De Jong WH, Jennen D, Keizers PH, Hodemaekers HM, Vermeulen JP, Bakker F, et al. Evaluation of adverse effects of resorbable hyaluronic acid fillers: determination of macrophage responses. Int J Mol Sci. 2022;23(13):7275. 10.3390/ijms23137275 35806280 PMC9266407

[156] Cox SE, Adigun CG. Complications of injectable fillers and neurotoxins. Dermatol Ther. 2011;24(6):524–36. 22515668 10.1111/j.1529-8019.2012.01455.x

[157] Lafaille P, Benedetto A. Fillers: contraindications, side effects and precautions. J Cutan Aesthet Surg. 2010;3(1):16–9. 10.4103/0974-2077.63222 20606987 PMC2890129

[158] Houseman ND, Taylor GI, Pan W-R. The angiosomes of the head and neck: anatomic study and clinical applications. Plast Reconstr Surg. 2000;105(7):2287–313. 10845282 10.1097/00006534-200006000-00001

[159] Cohen JL. Understanding, avoiding, and managing dermal filler complications. Dermatol Surg. 2008;34:S92–9. 10.1097/00042728-200806001-00020 18547189

[160] Weinberg MJ, Solish N. Complications of hyaluronic acid fillers. Facial Plast Surg. 2009;25(05):324–8. 10.1055/s-0029-1243081 20024874

[161] Sadowitz B, Seymour K, Gahtan V, Maier KG. The role of hyaluronic acid in atherosclerosis and intimal hyperplasia. J Surg Res. 2012;173(2):e63–72. 10.1016/j.jss.2011.09.025 22104612

[162] Brody HJ. Use of hyaluronidase in the treatment of granulomatous hyaluronic acid reactions or unwanted hyaluronic acid misplacement. Dermatol Surg. 2005;31(8):893–7. 10.1097/00042728-200508000-00001 16042932

[163] Menon H, Thomas M, D’silva J. Low dose of Hyaluronidase to treat over correction by HA filler – a case report. J Plast Reconstr Aesthet Surg. 2010;63(4):e416–7. 10.1016/j.bjps.2010.01.005 20133216

[164] Costerton JW, Cheng K-J, Geesey GG, Ladd TI, Nickel JC, Dasgupta M, et al. Bacterial biofilms in nature and disease. Annual Review of Microbiology. 1987;41: 435–464 10.1146/annurev.mi.41.100187.002251 3318676

[165] Hoyle BD, Jass J, Costerton JW. The biofilm glycocalyx as a resistance factor. J Antimicrob Chemother. 1990;26(1):1–5. 10.1093/jac/26.1.1 2211430

[166] Sadashivaiah AB, Mysore V. Biofilms: their role in dermal fillers. J Cutan Aesthet Surg. 2010;3(1):20–2. 10.4103/0974-2077.63257 20606988 PMC2890130

[167] Carlos-Fabuel L, Marzal-Gamarra C, Martí-Álamo S, Mancheño-Franch A. Foreign body granulomatous reactions to cosmetic fillers. J Clin Exp Dentist. 2012;4(4):e244. 10.4317/jced.50868 PMC3917632 24558563

[168] Reisberger E-M, Landthaler M, Wiest L, Schröder J, Stolz W. Foreign body granulomas caused by polymethylmethacrylate microspheres: successful treatment with allopurinol. Archiv Dermatol. 2003;139(1):17–20. 10.1001/archderm.139.1.17 12533157

